# CVD Risk Factors in the Ukrainian Roma and Meta-Analysis of Their Prevalence in Roma Populations Worldwide

**DOI:** 10.3390/jpm11111138

**Published:** 2021-11-02

**Authors:** Matea Zajc Petranović, Ashley Elizabeth Rizzieri, Dharshan Sivaraj, Nina Smolej Narančić, Tatjana Škarić-Jurić, Željka Celinšćak, Anita Stojanović Marković, Marijana Peričić Salihović, Julia Kalászi, Marianna Kalászi, John Q. Lin, Sanica Mehta, Jill Burleson, David A. Rizzieri

**Affiliations:** 1 Institute for Anthropological Research, Gajeva 32, 10000 Zagreb, Croatia; smolej55@gmail.com (N.S.N.); tanja@inantro.hr (T.Š.-J.); zeljka.celinscak@inantro.hr (Ž.C.); astojanovic@inantro.hr (A.S.M.); mpericic@inantro.hr (M.P.S.); 2 Division of Cellular Therapy, Duke University, 2400 Pratt Street, Durham, NC 27708, USA; Arizzieri@auburn.vcom.edu (A.E.R.); Ds311@stanford.edu (D.S.); juliiakalaszi@gmail.com (J.K.); kalaszimarianna@gmail.com (M.K.); sanicamehta72@gmail.com (S.M.); jill.burleson@duke.edu (J.B.); 3 School of Medicine, Stanford University, 291 Campus Drive, Stanford, CA 94305, USA; jqlin@stanford.edu

**Keywords:** Roma, Ukraine, population health, meta-analysis, cardiovascular health, risk factors

## Abstract

The Roma population suffers from severe poverty, social exclusion, and some of the worst health conditions in the industrialized world. Herein, we report on cardiovascular disease (CVD) risk factors in the Ukrainian Roma and present a meta-analysis of the prevalence of CVD risk factors in 16 Roma populations worldwide. The meta-analyses of CVD risk factors in Roma (*n* = 16,552) vs. non-Roma majority population of the same country (*n* = 127,874) included publicly available data. Ukrainian field survey included 339 adults of both sexes and outcomes of interest were hypertension, body mass index (BMI), smoking, education, and employment status. Furthermore, 35.7% of the Ukrainian Roma were hypertensive, 69.3% unemployed, and 48.4% never went to school. Ukrainian Roma women were more likely to be underweight and more prone to be hypertensive, with odds of hypertension increasing with age, BMI, and positive smoking status. Meta-analyses showed that, in comparison with non-Roma worldwide, the Roma bear significantly higher risk factor loads related to smoking (OR = 2.850), diabetes (OR = 1.433), abdominal obesity (OR = 1.276), and metabolic syndrome (OR = 1.975), with lower loads for hypertension (OR = 0.607) and BMI ≥ 25 kg/m^2^ (OR = 0.872). To conclude, the CVD risk factors which are more common in Roma than in the majority population may reflect their poor health-related behaviors and inadequate access to health education.

## 1. Introduction

The Roma population (Gypsies) is a transnational minority population of a common Indian origin present in most of the world countries. Their population is comprised of over 15,000,000 people, most of whom live in Europe (12,000,000). They are an example of a founder population with persistent, centuries-long socio-cultural isolation. Today, there are diverse groups that term Roma includes: Gypsies, Travellers, Manouches, Ashkali, Sinti, and Boyash/Bayash [1].

Although migratory throughout most of their history, the majority of Roma are no longer nomadic. However, despite having a permanent residence, they generally live separated and excluded from the majority communities in surrounding settlements, often in overcrowded and dilapidated housing with poor sanitation, and lacking adequate water and hygiene [2,3]. Further, Roma are generally poorly educated and many of them are early school-leavers [2]. The EU-MIDIS II study found that the highest proportion of Roma who did not complete any level of formal education were in Greece and Portugal (especially in the age group of over 45 years; more than 70% of Roma). High proportions were also found in Spain and Croatia, while a small proportion of Roma without any formal education were in the Czech Republic, Slovakia, and Bulgaria [2]. These conditions affect the health of the Roma population, resulting in poorer health status, shorter life expectancy, and higher infant mortality rates in comparison to surrounding majority populations [4]. Both communicable [5,6] and non-communicable diseases are also present in significantly higher prevalence among Roma populations [7,8,9,10,11]. Thus, concerted efforts initiated by European Union (EU) member countries and some international organizations have attempted to close the socioeconomic gaps between Roma populations and the rest of society [12,13,14].

The Roma in Ukraine, similar to the Roma in the rest of Europe, are a heterogeneous population of approximately 120,000 and 400,000 members, whose socioeconomic situation varies among different regions of the country [15]. They share the same problems as Roma in other countries: high rates of illiteracy and unemployment, poor living conditions, and a lack of adequate care and development assistance. Ukraine, although not being an EU member country, established a ‘Strategy on the Protection and Integration of the Roma national minority into Ukrainian Society up to 2020’ in 2013, along with a subsequent ‘National Action Plan (NAP) on Implementation of the Strategy’. Still, reports suggest that the integration of Roma into the wider society has not progressed [16]. Although the NAP gains some insight into the difficulties in providing Roma with adequate healthcare, little is known about the health status of this population in Ukraine. For example, there are just few sentences in the book published in 2013 that noted an increase in various cardiovascular and infectious diseases among the Roma population in Ukraine, and a problem of malnutrition [17].

In this study, we present the first known epidemiologic data on cardiovascular disease (CVD) risk factors in the Roma living in Ukraine. Furthermore, in order to investigate whether Roma as a population really bear higher burden for non-communicable diseases, we performed the first meta-analysis of the prevalence of various CVD risk factors (hypertension, overweight, obesity, central obesity, smoking, diabetes, and metabolic syndrome) in Roma compared to majority populations from 16 countries worldwide.

## 2. Materials and Methods

### 2.1. Ukrainian Sample

A medical aid mission focused on the Roma people was conducted during August 2016, in five villages (Batrad, Perechyn, Kholmok, Koson, and Velyka Dobron) located in the Zakarpattia region of Ukraine, by a team of healthcare providers and investigators from Duke University Medical Center. Data were extracted and analyzed from the medical charts/notes made during the care of these patients as well as their accompanying families. The Duke University Institutional Review Board approved this study prospectively with a waiver of informed consent, since this was an observational, non-interventional study using data collected as part of the standard care for the patients. The Zakarpattia region is in southwestern part of Ukraine and is the only Ukrainian region that has boundaries with four countries (Poland, Slovakia, Hungary, and Romania). It is inhabited by approximately 1255 thousand people, representing 3.3% of the total Ukrainian population (Ukrainian Census, 2001). A total of 47,587 members of Roma population live in Ukraine, approximately 14,000 of whom (29.4%) inhabit the Zakarpattia region. These 14,000 Roma in Zakarpattia represent 1.1% of the total Zakarpattia population (Ukrainian Census 2001). However, unofficial estimates indicate that there are between 120,000 and 400,000 of Roma in Ukraine, so if we translate the official estimates that approximately 30% of the total Roma in Ukraine inhabit the Zakarpattia region, there might be between 36,000 and 120,000 Romani [16].

The Zakarpattia region consists of “raions” (districts). Before 2020, there were 13 “raions” (now there are six “raions”), in three of which were placed our five villages: Berehove raion (among other villages, there are also villages Batrad and Koson), Uzhhorod raion (among other villages, there are also Velyka Dobron and Kholmok), and Perechyn raion (with Perechyn as its administrative center). The latter no longer exists; from 2020 onwards, it is a part of the Uzhhorod raion. The Census 2001 data reported that there were 1705 Roma in the Uzhhorod raion, 1695 Roma in the Berehove raion, and 138 Roma in the Perechyn raion. No official Ukrainian Census data are available on the size of the population of these five villages in 2001 or later, nor on the exact number of Roma in each of the five villages.

All Roma from the listed five villages were invited to come, to be examined free of any charge and the word was spread (and communication during the examination was accomplished) with the help of local volunteers who speak Ukrainian, Hungarian, and Romani language, as well as English. It was estimated that a written announcement and invitation would not make sense because a large number of Roma cannot read.

Health status and habits questionnaires were filled out, as part of the standard care provided during a medical aid trip led by co-authors of this work (D.A.R, D.S., J.K., M.K. and J.B.), by the health care team due to the high degree of illiteracy among participants. Anonymized data were obtained from a total of 337 adults (114 men and 223 women) aged 18 years and older during this outreach effort, plus two women older than 17.5 years. Participants were self-declared as members of the Roma population. All participants received the results of the measured variables along with appropriate consulting with a health provider. None of the participants had health insurance. According to data from the Ukrainian Census 2001, Roma respondents in this research represent approximately 9.6% of all the Roma from three Zakarpattia region districts: the Berehove raion, the Uzhhorod raion and the Perechyn raion, approximately 2.4% of the officially acknowledged Roma population in the Zakarpattia region, while compared to unofficial estimates, 0.94% of Roma in Zakarpattia at best and 0.28% at worst.

The study protocol incorporated the measurement of various health-related biometric traits and interviews. Each examinee participated in an interview covering health related issues, such as dietary and smoking habits, diagnosed and undiagnosed health problems, level of educational attainment, and employment status. Weight and stature were measured following standard International Biological Programme Protocol [18]. Weight was determined to the nearest 0.5 kg using a portable scale. Stature was measured to the nearest mm using a fixed stadiometer. The body mass index (BMI) was calculated as weight [kg]/stature [m]^2^. In order to use BMI as a surrogate marker for nutritional status, the WHO cutoff points to define the categories of nutritional status based on BMI were applied. Individuals with BMI values from 25.00 to 29.99 kg/m^2^ were categorized as overweight while those having BMI equal to or higher than 30.00 kg/m^2^ were classified as obese. Individuals with BMI values less than 18.50 kg/m^2^ were categorized as underweight. Blood pressure measurements were taken by an experienced medical team member on the aid trip through the use of a mercury sphygmomanometer. Measurements were taken in a sitting position and after a 10–15-min rest. The hypertensive group incorporated participants with previously diagnosed hypertension as well as those with a measured systolic blood pressure value of higher than 140 mmHg, or a diastolic blood pressure measure higher than 90 mmHg. Smoking status is shown as the proportion of people who smoke daily. The education level was presented as a categorical variable, defined according to the number of years of education (4 categories: none education vs. some education (1–8 years of compulsory education) vs. completed compulsory education (4 years of elementary school and 5 years of lower secondary school) vs. completed upper secondary school (2 years); or 2 categories: <9 vs. ≥9 years of education), and employment status was also presented as a categorical variable.

### 2.2. Meta-Analysis Sample

To compare the prevalence of several CVD risk factors (hypertension, overweightness, obesity, central obesity, smoking, diabetes, and metabolic syndrome) between the Roma and non-Roma worldwide (only Caucasians), electronic databases (PubMed, MEDLINE, and Science Direct) were searched up to August 2021 for similar studies. The keywords used for the search were: (Roma OR Roma population OR Romani OR Gypsy OR Traveller) AND (hypertension OR BMI OR overweight OR obesity OR central obesity OR abdominal obesity OR smoking OR diabetes OR T2D OR metabolic syndrome OR MetS). All languages were searched initially, but, with the one exception, only English language articles were finally selected for the meta-analyses. This exception were two articles written by Borissova et al. [19,20] in Bulgarian Cyrillic, because the data needed for the meta-analyses were in English in the abstracts. The primary search generated 264 potentially relevant articles, 52 of which met the inclusion criteria (Supplementary Figure S1 and Supplementary References). In addition, the references of the selected publications were searched for additional studies. Studies from 16 countries (listed in Table 1) were selected according to the following eligibility criteria: a study had to contain data on prevalence of at least one of the investigated CVD risk factors for both sexes and there had to be data on both investigated ethnic subsets (Roma and non-Roma) for the same included country. In case that there was more research found on the same ethnic group in the same country, sample sizes weighted according to the prevalence of the ethnic subgroup were summed for certain CVD risk factor. In some cases, the same database was used in several studies from the same country, but a certain variable (investigated CVD risk factor) was included only once in the meta-analysis.

The quality analysis of the studies included in the meta-analyses was assessed using a customized checklist containing nine criteria (parameters) for making judgements [21]. Three authors independently assessed the methodological quality of the included papers (M.Z.P., M.P.S., and A.S.M.) The criteria items were judged as follows: yes, no, and not applicable. Any disagreement was resolved by discussion. Only (yes) answers were given points (each answer signifies 1 point). The quality of meta-analysis was expressed as overall percentage of positive (yes) answers.

### 2.3. Statistics

Differences between sexes in CVD risk factors prevalence were tested using Fisher’s exact test and Pearson’s Chi square test. A multivariate logistic regression model tested the combined effect of several risk factors on hypertension using following variables: age, sex, education, employment status, nutritional status assessed through BMI, and smoking status. The analyses were performed by SPSS 21.0 statistical package for Windows (SPSS Inc., Chicago, IL, USA), with statistical significance set at *p* < 0.05.

In order to investigate possible differences in prevalence of CVD risk factors between Roma and non-Roma populations, odds ratios (ORs) and 95% CIs were employed. Heterogeneity between studies was assessed by the I^2^ statistics. Publication bias was tested by Begg’s and Egger’s tests. All statistical analyses were carried out using STATA version 17 (Stata Corporation, College Station, TX, USA).

To identify potential influential studies (countries), we calculated the effects estimates (ORs) by removing an individual study each time and then checked if the overall significance of the estimate or of the heterogeneity statistics was altered. Due to the composite nature of each country’s sample, cumulative and meta-regression analysis could not be assessed.

### 2.4. Supplementary Meta-Analyses (Confirmation of Representativeness of Roma Samples)

The authors are aware that there may be concerns about whether the samples included in the meta-analyses are representative of the Roma populations in their countries, and thus whether they are informative for the Roma population. Thus, we decided to conduct the additional meta-analyses of, what researchers claim to be, the representative sample of Roma population from six European countries (Bulgaria, Czech Republic, Greece, Romania, Slovakia, and Spain) and the representative sample of general population from respective countries. The Roma were participants in the European project “Health and the Roma Community, Analysis of the Situation in Europe” (the UNDP/WB/EC research), while general population data were provided by the Eurostat dataset. These publicly available data enabled five meta-analyses of CVD risk factors: hypertension, diabetes, smoking, BMI ≥ 25.00 kg/m^2^, and obesity (BMI ≥ 30.00 kg/m^2^) [22]. The prevalence of hypertension and diabetes mellitus was taken from the Eurostat EHIS folder Health status—historical data (2008), while the prevalence of nutritional status categories and smoking from the EHIS folder Health determinants—historical status (2008).

The Roma from the UNDP/WB/EC research consisted of 7604 people of all age, both men and women, selected to be representative to obtain statistically reliable data that can be extrapolated to the whole Roma community. We analyzed only data of 4575 adult Roma (16+ year-olds). Due to different sample sizes in different countries, the UNDP/WB/EC researchers weighted the figures of each country to make them comparable.

The Eurostat dataset comprised 59,120 people aged 16 and above who participated in the European Health Interview Survey (EHIS) wave 1, conducted in 2008. A list of countries and the prevalence of five investigated CVD risk factors and population sizes are given in Supplementary Table S2.

## 3. Results

Participant characteristics are described in Table 2 and encompass age, sex, BMI, smoking status, years of education, employment status, and presence of hypertension. A total of 339 Ukrainian Roma (114 men, 225 women) was sampled with a median age of 38 years. In all, 327 (96.5%) of the Ukrainian Roma received less than nine years of compulsory education, and 236 (69.6%) were currently unemployed (more women than men, *p* < 0.0001). A total of 208 individuals (61.4%) of the population were smokers, with an average cumulative cigarette consumption of 8.8 pack-years. Undernutrition measured by means of BMI was found in 12.7% of Roma (2.5 times was more frequent in women than in men), while overweight was found in 20.7% and obesity in 13.9% of the Roma (more men than women were overweight, while more women than men were obese, *p* = 0.005). Hypertension was found in 121 (35.7%) individuals and hypertensives were more likely to be older (33.04 vs. 44.81 years, *p* < 0.0001) and to have elevated mean BMI (22.39 vs. 26.54 kg/m^2^, *p* < 0.0001). Overweight and hypertension were most frequently present in the Roma aged 25–54 years when compared to younger and older age groups, while smoking was more prevalent in younger people (up to 54 years of age) than in the older population (Supplementary Material, Table S1).

The association of hypertension with cardiovascular disease risk factors was evaluated by comparing the prevalence of each risk factor between hypertensive and non-hypertensive Ukrainian Roma, i.e., sex, age, smoking status, obesity, unemployment, and less than 9 years of education (Table 3). Female sex (OR = 1.90, 95% CI = 1.07 to 3.38, *p* < 0.05), age of 45 years and older (OR = 6.14, 95% CI = 3.50 to 10.75, *p* < 0.0001), smoking (OR = 3.20, 95% CI = 1.81 to 5.69, *p* < 0.0001), and BMI ≥ 30 kg/m^2^ (OR = 8.92, 95% CI = 3.95 to 20.13, *p* < 0.0001) were significantly associated with hypertension in the Ukrainian Roma, while the level of education and employment status did not.

**Figure 1:**
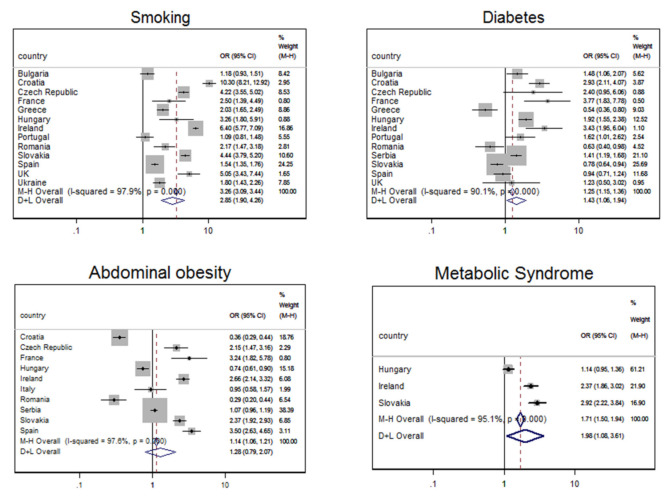
Forest plot of the association between the Roma ethnicity and higher odds ratio (OR) for the prevalence of various CVD risk factors (smoking, diabetes, abdominal obesity, and metabolic syndrome), compared to the majority population. Results are stratified by country.

**Figure 2:**
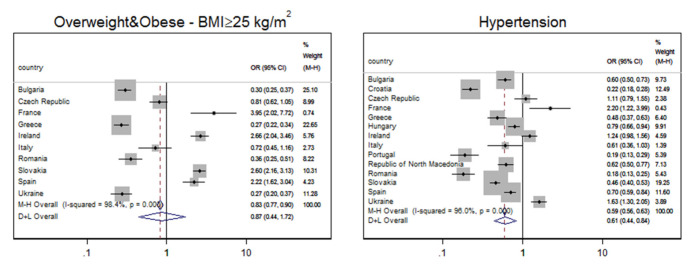
Forest plot showing an association between the Roma ethnicity and lower odds ratio (OR < 1) for the prevalence of BMI ≥ 25 kg/m^2^ and hypertension, compared to the majority population. Results are stratified by country.

The results of meta-analyses for smoking, diabetes, abdominal (central) obesity, and metabolic syndrome are presented in Figure 1**,** while meta-analyses for BMI ≥ 25 kg/m^2^ and hypertension are given in Figure 2. The variables were grouped this way because of the odds ratio results: Figure 1 shows variables for which ORs were significantly higher than 1 in Roma compared to the majority populations, while Figure 2 shows variables for which ORs in Roma were significantly lower than in majority populations. The quality of studies included in meta-analyses is evaluated in Supplementary Table S3; 95.4% of the included papers fulfilled all quality criteria.

In all thirteen countries analyzed, smoking was more prevalent in Roma than in non-Roma majority population, heterogeneity was high (I^2^ = 97.9%), and the OR for random effect model amounted to 2.850 (95% CI 1.904–4.264, *p* < 0.001). Diabetes was more prevalent in Roma than in non-Roma majority population in nine of the thirteen analyzed countries (exceptions were Greece, Romania, Slovakia, and Spain). Heterogeneity was also high (I^2^ = 90.1%) and the OR for random effect model amounted to 1.433 (95% CI 1.061–1.936, *p* < 0.001). Higher OR for abdominal obesity in Roma than in non-Roma was also detected (I^2^ = 97.6%, DerSimonian-Laird OR = 1.276, 95% CI 0.787–2.070, *p* < 0.001), just as for metabolic syndrome (I^2^ = 95.1%, DerSimonian-Laird OR = 1.975, 95% CI 1.082–3.606, *p* < 0.001).

On the other hand, odds for the Roma to have hypertension or BMI ≥ 25 kg/m^2^ were lower than in the non-Roma population. The overall OR to have BMI ≥ 25 kg/m^2^, despite individual ORs > 1 in four of the investigated ten countries, was lower in Roma in comparison with non-Roma population (I^2^ = 98.4%, DerSimonian-Laird OR = 0.872, 95% CI 0.442–1.721, *p* < 0.001). Individual ORs for hypertension in four of the investigated fourteen countries were also >1, but the overall OR was 0.607 (I^2^ = 96.0%, DerSimonian-Laird 95% CI 0.440–0.838, *p* < 0.001).

Neither Begg’s test nor Egger’s test showed a publication bias in any of the conducted meta-analyses. The meta influential analysis revealed that no single study (country) was responsible for the overall significance of the estimates (as shown in the Supplementary Material, Figures S2–S19). Meta-analysis for obesity (BMI ≥ 30 kg/m^2^) was also conducted but no significant differences between the Roma and non-Roma populations were found (as shown in the Supplementary Material, Figure S20).

Results similar to those presented above were obtained when meta-analyses were conducted between a representative sample of ethnic Roma (participants in the European project “Health and the Roma Community, Analysis of the Situation in Europe” from Bulgaria, Czech Republic, Greece, Romania, Slovakia and Spain), and a representative sample of the majority population (the EHIS 1 population from the Eurostat data). The Roma population had significantly higher odds ratio (OR) of having three CVD risk factors (obesity, smoking, and diabetes), compared to the majority population (Supplementary Figure S21), and significantly lower odds ratio for hypertension and BMI ≥ 25.00 kg/m^2^ (Supplementary Figure S22). Begg’s test showed no publication bias in any of the conducted meta-analyses, while Egger’s test showed bias in the obesity meta-analysis. The meta influential analysis revealed that no single study (country) was responsible for the overall significance of the estimates (data not shown in the Supplementary Material).

## 4. Discussion

Cardiovascular diseases (CVDs) are the major cause of deaths globally (31% of all cases), >75% of which occur in low- and middle-income countries. Persons at risk of CVD can have raised blood pressure, elevated blood glucose and lipids levels, increased abdominal circumference, or can be overweight or obese [23,24]. Identifying those with the highest risk of CVDs and giving them appropriate treatment and counselling can prevent premature deaths [25]. All parameters that indicate CVD risk can be easily measured and determined in primary care facilities, even as basic as the medical aid station provided by the Duke team visiting the Ukrainian Roma. However, for this to be possible, individuals must have access to basic health care. We obtained local approval for our offer of care from the near-by city medical official who visited our aid station and was supportive of our efforts; however, there was no plan known to us for continued outreach to this population other than the annual mission provided by this study’s co-author (JB). In our experience providing care during our aid trip, it was rare that a Roma person indicated seeking prior health care.

Regardless of the economic level of the country in which Roma live, this minority population is economically disadvantaged. The poverty of the Roma population is a result of their low level of education associated with inability to find a regular job, discrimination, and overwhelming social exclusion. The causes of these are twofold: the prejudices of majority populations and their hostility towards the Roma, but also the persistence of Roma to maintain their cultural identity (resistance to economic and social trends) [26]. In many countries, Roma are prevented from using healthcare because they cannot afford to pay health insurance contributions, either because they are not formally employed (or not registered in employment agencies), or because they do not have identification documents [17].

Our sample consisted of mostly young (68% were younger than 44 years) and practically illiterate (48.4% had none education and 48.1% did not complete compulsory education which lasts 9 years) people, who rarely had a permanent job (only 4.1%) and were without any health insurance. This situation is similar to that in other Roma populations as a small number of Roma attending school or with a high level of illiteracy, although lower than in Ukraine, was detected in Croatia [27], but also in other European and EU member countries [26,28,29], while inequality in healthcare access was reported in the Italian [30,31], the Croatian [27], and the Spanish Roma [32]. Review paper by McFadden et al. [33], based on the analysis of 99 studies from 32 countries, also provided evidence that Gypsy, Roma, and Traveller populations across Europe face barriers to healthcare access and have low literacy levels. The situation in health insurance availability has actually been changing in some EU member countries; 2019 analysis showed that 95% to 98% of Roma in Spain, Portugal, and Slovakia were covered either by the national basic health insurance scheme or additional insurance [26]. Still, the same survey indicated that this was the case for only 45% of Roma in Bulgaria and 54% of Roma in Romania.

The high prevalence of smoking in the Ukrainian Roma is very similar to the findings of the majority Ukrainian population in which also approximately two thirds of the participants smoked [34]. Malnutrition was found 2.6 times as often in the Ukrainian Roma women than in men (16.0% vs. 6.1%), and in total one in eight Roma had BMI < 18.5 kg/m^2^ (12.7%). This is not surprising since Roma often suffer from hunger: in 7% of the households surveyed in EU-MIDIS II survey from 2016 (conducted in Bulgaria, Czech Republic, Greece, Spain, Croatia, Hungary, Romania, and Slovakia), at least one Roma person goes to bed hungry four times or more a month [26]. Of the investigated EU member countries, the situation is the worst in Croatia (17%) and Greece (13%). We also want to emphasize that the difference in the prevalence of nutritional status categories (underweight, normal weight, overweight, and obese) between the sexes in Ukrainian Roma and in Croatian Bayash Roma study from 2006 was practically the same [35]; in both Roma populations, although being >600 km apart, there were more underweight women than men, an approximately equal proportion of men and women had a normal weight, more men than women were overweight, while more Roma women than Roma men were obese.

A higher prevalence of most of the investigated risk factors for non-communicable diseases was found in Roma populations in different countries when compared to majority populations [36,37,38,39,40,41]. In the review paper from 2000, authors reported limited evidence for increased morbidity from non-communicable diseases, because little (only one paper) was available on that topic [7]. Dobranici et al. [9] focused their review on Roma in central-eastern Europe, specifically the idea that risk factors for CVD in shortening life expectancy in Roma could not be determined due to a paucity of reliable data. Cook et al. [42], in a systematic review of the epidemiological literature related to the Roma population, reported evidence for significantly higher mortality risk for Roma compared to non-Roma, but also stated the problem of insufficient number of studies on non-communicable diseases to make firm conclusions. More recently, Papon et al. [10] revised various CVD risk factors (hypertension, dyslipidemias, obesity, diabetes, and abdominal obesity) in Roma from eight countries, while Nunes et al. [11] revised prevalence of diabetes in Roma from five countries. Again, no firm conclusion could be drawn due to the small number of published literature and small number of participants.

To our knowledge, this is the first meta-analysis of CVD risk factors comparing Roma (16,552) and same country non-Roma majority populations (127,874) in 16 countries worldwide. Higher odds for smoking, diabetes, abdominal obesity, and metabolic syndrome in Roma compared to non-Roma are consistent with the results of most research conducted to date on the subject. Similar results were obtained even when only a representative sample of Roma and a representative sample of the majority population from six European countries were included in the meta-analyses. Still, high heterogeneity limits the interpretation of our meta-analytic results. Whether it is based on data or design, measurement instruments, or analytical methods, precise answers to broad meta-analytic questions about subjective issues may be difficult to achieve when heterogeneity is high [43].

Chances to have hypertension are lower if the person is Roma than if they are non-Roma. Although we would have expected that Roma populations, who have really low socioeconomic status, have higher blood pressure, especially since level of education defines most vulnerable groups [44], it seems that further research is required to identify factors which contribute to reduction in the risk for hypertension in Roma. For starters, more attention might be paid to investigate the possible protective role of nutrition on hypertension, as data on food consumption in Roma are scarce. In some studies, results indicated that the Roma have inferior diet diversity compared to the non-Roma [45,46,47] or that they differ in their energy intake [48], but the most interesting results showed Kozubik and colleagues [49] who compared data on Roma in eastern Slovakia from 2013 and from 1775: their conclusion was that eating habits of contemporary Roma were still similar to those of two centuries ago. Therefore, this finding could also indicate early stage of epidemiological transition present in this unprivileged population.

A significant difference in abdominal circumference between Roma and non-Roma is also concerning, because it is likely that both groups have too much ectopic body fat. There seem to be general trend of secular waist circumference increasing beyond that expected by BMI [50,51].

In addition to environmental and lifestyle factors, it seems that genetic reasons are behind the differences in the frequency of CVD risk factors between Roma and non-Roma populations. Recent research has shown that the increased prevalence of diabetes in Czech Roma [52], as well as the increased average BMI and waist circumference [53] and the reduced prevalence of hypertension [54] in the Hungarian Roma population, may be associated with different frequencies of the risk alleles in genes associated with the development of these phenotypes. Some of the SNPs investigated in these studies have been identified as potentially useful for assessing genetic risk score in the Roma population. On the other hand, also in Hungarian Roma, SNP-based genetic risk score modeling did not show increased genetic susceptibility of the Roma population to type 2 diabetes [55], or smoking [56].

### Strengths and Limitations

This is the first research on the socio-economic status and health of the Roma from Zakarpattia in Ukraine, and the first meta-analysis of seven CVD risk factors between Roma and non-Roma. Despite the fact that we analyzed a relatively small sample taking into account the total number of Roma in Ukraine, we have no reason to believe that their results differ from the rest of Ukrainian Roma population. The low socio-economic status and poor health of Ukrainian Roma are in line with the findings of other studies on the Roma population in Europe; even in high-income countries, such as Spain and Greece, compared to the non-Roma population, Roma have poorer health, which is closely linked to social determinants of health.

Still, there is a possibility that the Ukrainian Roma who came to our field clinic may have biased the sample on two grounds: (1) based on the villagers’ mistrust and acceptance of outside care by foreigners; and (2) Roma with medical problems would be more motivated to come seek care than the healthy ones, especially since they did not have health insurance and we offered medical examination for free. Furthermore, bLood pressure of the Ukrainian Roma was not measured exactly according to the recommendations; they were measured once and were recommended to be measured twice, the second measurement after a 15-min rest. Providing care at the improvised medical clinic, many of whom waited for hours to be examined, limited our possibilities. This factor does present limitations to the reported hypertension prevalence.

Our meta-analyses are not registered in any registry of systematic reviews.

## 5. Conclusions

To conclude, in a sample of Roma from Ukraine, 96% of whom did not complete compulsory education, the prevalence of hypertension, smoking, malnutrition, and obesity is a matter of concern. This survey yielded similar results on Roma life as other Roma studies conducted in other countries. Our comprehensive meta-analysis of the investigated CVD risk factors showed that Roma from Ukraine and 15 other countries were more prone to smoke and develop diabetes or abdominal obesity, while less prone to hypertension, but high heterogeneity limits the interpretation of these results. Nevertheless, studies such as ours can be useful as an impetus for the development of a targeted, population-specific intervention program to improve living conditions and for the prevention of CVD risk factors in Roma population. Trends observed in Roma populations across Europe are an indicator of a certain degree of epidemiological transition (in socio-economic status, age structure), cultural factors (smoking, diet), and perhaps even genetic background, which all together contribute to the typical Roma structure of risk factors for the development of cardiovascular diseases.

## Figures and Tables

**Table 1:** Characteristics of 52 studies included in the meta-analysis presented by countries. Age data in this table are not presented uniformly because they were reported differently in the papers: some authors preferred age range, others mean age (SD) or just reported that subjects were over a certain age. We divided subjects from each of the 16 countries in two population groups: group one were Roma, while group two were non-Roma. Each study group consisted of both females and males. Each of the investigated CVD risk factors is presented as a prevalence (percentage) and not in absolute numbers. Complete references of these studies are given in the Supplementary file.

Country	Paper	Population	Additional Data	Sample Size	Sex Ratio (m/f)	Age Range (yrs)	Mean Age	Hypertension (BP ≥ 140/90 mm Hg)	Overweight & Obese (BMI ≥ 25 kg/m^2^)	Obese (BMI ≥ 30 kg/m^2^)	Central Obesity (WHR m > 0.90, f > 0.85 or WC m > 102 cm, f > 88 cm)	Smoking	Diabetes (Previously Diagnosed or/and Fasting Glucose ≥ 7 mmol/L)	MetS
Bulgaria	ˆ Fundación Secretariado Gitano 2009	Roma	Participants in the European project “Health and the Roma Community, Analysis of the Situation in Europe”	548		15+		32.5%	41.7%	14.2%		46.1%	9.3%	
Bulgaria	Naydenov St et al. 2011	non-Roma Bulgarians	a cross-sectional study conducted in 2010 in 4 large Bulgarian cities	1535	689/846	14–95	58.9 ± 16.4						5.7%	
Bulgaria	Borissova A-M et al. 2015a	non-Roma Bulgarians	not comprehensible, article written in cyrillic	1967	917/1050	20–80		38.9%						
Bulgaria	Borissova A-M et al. 2015b	non-Roma Bulgarians	not comprehensible, article written in cyrillic	1958	912/1046	20–80			70.2%	33.2%				
Bulgaria	Hadjiev et al. 2003	non-Roma Bulgarians	prospective epidemiological study carried out in the University city of Stara Zagora	500	200/300	50–79		65.8%		58.3%		41.9%	8.8%	
Croatia	Zeljko et al. 2013	Roma	rural population from Baranja and Međimurje	430	151/279	18–84	41.3 ± 15.1	26.3%	49.1%	20.9%	37.4%	70.0%	16.0% ^θ^	
Croatia	Musić Milanović et al. 2010	non-Roma Croatians	the Croatian Adult Health Cohort Study 2008	3229	1015/2214	18+	NA			31.3%	62.7% ^¥^			
Croatia	Ivičević Uhernik & Erceg 2010	non-Roma Croatians	the Croatian Adult Health Cohort Study 2008	3229	1015/2214	18+	NA	61.6%						
Croatia	Samardžić & Vuletić 2010	non-Roma Croatians	the Croatian Adult Health Cohort Study 2008	2837		18+	NA					18.5%		
Croatia	Metelko et al. 2008	non-Roma Croatians	a representative sample from the First Croatian Health Project 1994–1997	1635	821/814	18–65	41.49 ± 12.18						6.1%	
Czech Republic	Adamkova et al. 2015	Roma	South Bohemia Region, snowball sampling	302	152/150	18+	NA						26.8%	
Czech Republic	Adamkova et al. 2015	non-Roma Czechs	non-Roma individuals selected using snowball sampling from South Bohemia Region	78		18+	NA						6.4%	
Czech Republic	Šedova et al. 2015	Roma	South Bohemia Region, snowball sampling	302	152/150	18+		24.3%	61.8%	31.9%	31.8% ^~^			
Czech Republic	Šedova et al. 2015	non-Roma Czechs	quota selection method (gender representative), non-Roma population for comparison with Roma, from South Bohemia Region	298	148/150	18+		17.8%	57.4%	17.1%	17.8% ^~^			
Czech Republic	Urban & Kajanova 2011	Roma	southern part of Czech Republic and Moravian-Silesian Region	164	52/112	15+						87.5%		
Czech Republic	Vanova et al. 2018	non-Roma Czechs	a representative sample of the Czech Republic’s adult population	1806	880/926	15+						25.2%		
Czech Republic	ˆ Fundación Secretariado Gitano 2009	Roma	Participants in the European project “Health and the Roma Community, Analysis of the Situation in Europe”	681		15+		17.2%	47.9%	20.2%		58.4%	8.5%	
France	Papon et al. 2017	Roma	Manouche community of Pau, Southwestern France	50	17/33	37.3 ± 13.04 (1 individual was 65+)	37.3 ± 13.4	32%	78%	38%	64%	34%	18%	
France	ObEpi-Roche 2012	non-Roma French	representative sample from whole France	25,714	12,214/13,500	18+		17.6%	47.3%	15%	35.4% ^¥^	17.1%	5.5%	
Greece	ˆ Fundación Secretariado Gitano 2009	Roma	Participants in the European project “Health and the Roma Community, Analysis of the Situation in Europe”	426		15+		16.3%	32.7%	9.4%		56.3%	6.7%	
Greece	Gikas et al. 2013	non-Roma Greeks	community-based cross-sectional study conducted among residents of Saronikos municipality (Attica region)	2636	1306/1330	20–95	50.5 ± 17.8	28.8%	64.1%	21.6%		38.9%	12.0%	
Hungary	Hidvegi et al. 2012	Roma	city of Gyor and surrounding area	77	35/42	20–70	46.9 ± 10.6	61% *			51.9% ^~^		18.2%	50.6%
Hungary	Paulik et al. 2011	Roma	2009 survey of Roma and non-Roma residents living in and around the city of Szeged in southern Hungary	83	42/41	16–70						72.3%		
Hungary	Paulik et al. 2011	non-Roma Hungarians	2009 survey of Roma and non-Roma residents living in and around the city of Szeged in southern Hungary	126	51/75	16–70						44.4%		
Hungary	Kosa et al. 2014	Roma	Hungarian counties (Hajdu-Bihar and Szabolcs-Szatmar-Bereg)	646	393/253	20–64		40.4% *			60.7% **		27.1%	36.4%
Hungary	Kosa et al. 2014	non-Roma Hungarians	a representative sample of the Hungarian population	1542	732/810	20–64		48.4% *			69.7% **		15.6%	35.0%
Hungary	Nagy et al. 2017	Roma	Roma from counties in Northeast Hungary	1152	465/687	20–69	41.4			26.6%				
Hungary	Nagy et al. 2017	non-Roma Hungarians	the General Practitioners’ Morbidity Sentinel Stations Program in 2006	1743	818/925	20–69	46.0			30.9%				
Ireland	Slattery et al. 2011	Irish Travellers	a sample population of Travellers along the Irish Atlantic seaboard	354	127/227		37 ± 11.21	39%	80%	47%	61%		5.9%	39.3%
Ireland	AITHS 2010	Irish Travellers	participants in the All Ireland Traveller Health study	2038	887/1151	18+						52.2%		
Ireland	Waterhouse et al. 2009	non-Roma Irish	a cross sectional study to investigate the prevalence of the MetS in participants attending an Executive Health Screening program at the Department of Preventative Medicine, Blackrock Clinic, Dublin, Ireland	1716	1026/690	32–78						11.4%	1.8%	21.4%
Ireland	Balanda et al. 2013	non-Roma Irish	participants in the survey of lifestyle attitudes and nutrition (SLAN) 2007	2099		18+							5.0%	
Ireland	Barron et al. 2014	non-Roma Irish	participants in the survey of lifestyle attitudes and nutrition (SLAN) 2007	2174		18+		34.1%						
Ireland	Healthy Ireland Survey 2015	non-Roma Irish	The Healthy Ireland Survey is conducted as part of Healthy Ireland, the National Framework for action to improve health and wellbeing of people living in Ireland	6142		15+			60%	23%	37% ^¥^	19%		
Italy	Gualdi-Russo et al. 2009	Roma	Balkan Roma, immigrants from southeastern Europe	70	32/38		males 37.3 ± 14.9; females 38.1 ± 14.4	28.6%	55.7%	21.4%	34.3%			
Italy	Donfrancesco et al. 2008	non-Roma Italians	Forty GPs from the Italian Association of General Practitioners (SIMG) and homogeneously distributed across the country screened 56 patients aged 35–74 years, randomly selected by the National Institute of Health (ISS) from each GP’s patient list in order to have 7 patients in each age decade and sex (35–44, 45–54, 55–64, 65–74 years)	2090	1044/1046	35–74		39.6%	63.6%	22.4%	35.5% ^¥^			
Portugal	ˆ Fundación Secretariado Gitano 2009	Roma	Participants in the European project “Health and the Roma Community, Analysis of the Situation in Europe”	245		15+		11.2%	54.7%	14.0%		26.9%	9.2%	
Portugal	Alves et al. 2015	non-Roma Portuguese	EPIPorto study	1550	578/972	35–65	50.8 ± 8.2	39.6%		23.4%	30.6%	25.5%	6.0%	
Republic of North Macedonia	Pavlovski 2009	Roma	Roma from 8 settlements in Macedonia	636	268/368	18+		40.3%						
Republic of North Macedonia	Rexhepi et al. 2018	non-Roma Macedonians	subjects selected at random from the primary healthcare register, to constitute a representative sample of a population in the district of Tetovo	630	310/320	18+	43.81 ± 16.01	52.1%						
Romania	Bartos et al. 2013	Roma	urban and rural (400 living in Bucharest, others of rural origin)	911	293/618	18–83	45 ± 15.3	31.2%		34.6%	51.1%	37.3%	14.2%	
Romania	Enache et al. 2016	Roma	mixed urban and rural (Calarasi county)	180	61/119	18–85		48.9%	58.3%	31.7%	72.8% **	45.6%	11.7%	
Romania	Enache et al. 2016	non-Roma Romanians	mixed urban and rural (Calarasi county)	164	56/108	18–85		64%	69.5%	32.3%	80.5% **	23.1%	14.6%	
Romania	ˆ Fundación Secretariado Gitano 2009	Roma	Participants in the European project “Health and the Roma Community, Analysis of the Situation in Europe”	1592		15+		17.5%	43.4%	16.9%			6.9%	
Serbia	Beljić-Živković et al. 2010	Roma	11 urban and 8 rural settlements	1465	512/953	18+	42.42 ± 15.69				41% ****		11.1%	
Serbia	Janković et al. 2019	non-Roma Serbians	participants in the 2013 National Health Survey	13,100	5985/7115	20+	51.0 ± 17.4					50.3%	8.1%	
Serbia	Maksimović et al. 2016	non-Roma Serbians	participants in the 2013 National Health Survey	12,460	6007/6453	20+	48.8 ± 17.0		58.8%	22.4%	39.8%			
Slovakia	Vozarova de Courten et al. 2003	Roma	rural (village Zlate Klasy)	156	70/86	30+	47	49%		65%	38%	42%	30%	20%
Slovakia	Vozarova de Courten et al. 2003	non-Roma Slovakians	rural (village Zlate Klasy)	501	230/271	30+	52	43%		30%	20%	21%	10%	4%
Slovakia	Krajcovicova-Kudlackova et al. 2002	Roma	western Slovakia (Gbely region)	149	57/92	20–60	40.7 ± 0.8	16%		36%		50%	17%	
Slovakia	Krajcovicova-Kudlackova et al. 2002	non-Roma Slovakians	western Slovakia (Gbely region)	197	96/101	19–60	40.1 ± 1.0	10%		22%		20%	16%	
Slovakia	Krajcovicova-Kudlackova et al. 2004	Roma	volunteers, apparently healthy young Gypsies from regions Dunajska Streda and Skalica	122		19–35		4%	52%	20%		55%	7%	
Slovakia	Krajcovicova-Kudlackova et al. 2004	non-Roma Slovakians	volunteers, randomly selected apparently healthy young Slovakians	137		19–35		3%	28%	8%		25%	6%	
Slovakia	Babinska et al. 2013	Roma	participants in the Hepa-Meta Study (from settlements in the Kosice region)	452	159/293	18–55	34.7 ± 9.14	27.2% *			55.8% **	47.3%	9.1% ***	
Slovakia	Babinska et al. 2013	non-Roma Slovakians	control population for participants in the Hepa-Meta Study (from settlements in the Kosice region)	403	185/218	18–55	33.5 ± 7.4	28.0% *			43.9% **	18.8%	7.4% ***	
Slovakia	Petrikova et al. 2018	Roma	participants in the Hepa-Meta Study (from settlements in the Kosice region)	442	35.2%/64.8%	18–55	34.7							29.6%
Slovakia	Petrikova et al. 2018	non-Roma Slovakians	control population for participants in the Hepa-Meta Study (from settlements in the Kosice region)	399	45.9%/54.1%	18–55	33.5							20.1%
Slovakia	Fedačko et al. 2014	Roma	eastern Slovakia	420	159/261		34.7		54.3%	27.2%				
Slovakia	Fedačko et al. 2014	non-Roma Slovakians	eastern Slovakia	382	181/201		33.5		44.1%	13.5%				
Slovakia	Urban & Kajanova 2011	Roma	eastern Slovakia	149	38/111	15+						40.3%		
Slovakia	Sudzinova et al. 2015	Roma	patients who underwent routine elective CAG in the East Slovakian Institute for Cardiac and Vascular Diseases, Kosice, Slovakia, in the years 2001–2011	167	118/49		52.1 ± 8.3	65.7%				35.2%	27.7%	
Slovakia	Sudzinova et al. 2015	non-Roma Slovakians	patients who underwent routine elective CAG in the East Slovakian Institute for Cardiac and Vascular Diseases, Kosice, Slovakia, in the years 2001–2011	649	398/251		57.8 ± 7.4	81.9%				9.3%	31.1%	
Slovakia	ˆ Fundación Secretariado Gitano 2009	Roma	Participants in the European project “Health and the Roma Community, Analysis of the Situation in Europe”	336		15+		17.7%	43.7%	17.9%		53.2%	6.0%	
Spain	Jimenez-Sanchez et al. 2013	Roma	participants in the National Health Survey in the Romany population 2006	993	466/527	16+		15.1%		21.2%		40.1%	5.6%	
Spain	Jimenez-Sanchez et al. 2013	non-Roma Spanish	participants in the Spanish National Health Survey 2006	16,079	7900/8179	16+		20.2%		14.6%		30.2%	6.0%	
Spain	Poveda et al. 2014	Roma	people from Greater Bilbao region (Basque County)	215	81/134		males 34.9 ± 11.35; females 34.27 ± 11.59		77.3%	51.7%	62.8%			
Spain	Aranceta-Bartrina et al. 2016	non-Roma Spanish	participants in the ENPE study (Spanish acronym for the Nutritional Study of the Spanish Population)	3966	1921/2045	25–64			60.9%	21.6%	33.4% ^¥^			
UK	Parry et al. 2007	Gypsies and Travellers of UK or Irish origin	five locations in England (Sheffield, Leicester, Norfolk, London and Bristol)	260	88/172	16–87	38.1 ± 15.4					58.1%	4%	
UK	Parry et al. 2007	age and sex matched comparison sample of non-Gypsies or non-Travellers from rural communities	people from rural communities, deprived inner-city White residents and ethnic minority	260	88/172	16–82	38.4 ± 15.2					21.5%	4%	
Ukraine	this study	Roma		339	114/225	17–85	37.7 ± 15.3	39.2%	34.5%	13.9%		61.4%		
Ukraine	Chagarna & Andreeva 2014	non-Roma Ukrainians	participants in the survey “Health and Well-Being in Transition Societies” (2000)	1635	720/915	15–92			44%			28.8%		
Ukraine	Pradhan D 2014 (Master Thesis)	non-Roma Ukrainians	data from the “Ukraine Household Survey”, conducted in the 8 regions of Ukraine (Kyiv, Autonomous Republic of Crimea, Vinnytsia, Rivne, Lviv, Dnipropetrovsk, Luhansk and Odessa) in the year 2009 by the Ukraine Centre for Economic and Political Studies	1342	1034/309	18–65						68.9%		
Ukraine	Pradhan D 2014 (Master Thesis)	non-Roma Ukrainians	data from the “Ukraine Household Survey”, conducted in the 8 regions of Ukraine (Kyiv, Autonomous Republic of Crimea, Vinnytsia, Rivne, Lviv, Dnipropetrovsk, Luhansk and Odessa) in the year 2009 by the Ukraine Centre for Economic and Political Studies	3425	1531/1894	18–65		27.6%						
Ukraine	Bilovol et al. 2017	non-Roma Ukrainians	randomly selected patients, recruited while visiting the out-patient unit of Kharkiv National Medical University due to any reason, except acute heart pathology, provided they had not had heart rate disorders in their past medical history	398		37–56	41.4 ± 2.3	35.7%	65.9%	23.9%				

ˆ In Fundación Secretariado Gitano 2009 study, a total of 7604 Roma of all ages and from seven EU countries were selected to be representative to obtain statistically reliable data that can be extrapolated to the whole Roma community. Due to different sample sizes in different countries, the researchers weighted the figures of each country to make them comparable. Data on Roma in Spain are not taken from this publication as they are presented in a study by Jimenez-Sanchez et al. 2013. A symbol “+” in the “Age range (yrs)” column means “and above”. * BP ≥ 130/85 mm Hg; ** WC ≥ 94 cm for men, ≥80 cm for women; ^~^ WC ≥ 103 cm; ^±^ WC ≥ 102 cm men, WC ≥ 88 cm women; *** International Diabetes Federation 2005-glucose ≥ 5.6 mmol/L; **** assessed by visual inspection; ^¥^ MetS by IDF criteria; In Krajcovicova-Kudlackova et al. (2002) study, diabetes was defined as glucose > 6.1 mmol/L and hypertension as sistol > 140 mmHg, ^θ^ GLU > 6.00 mmol/L.

**Table 2:** Demographic, socio-economic, and health-related characteristics of the Ukrainian Roma population with sex differences.

	Total		Male		Female		Male vs. Female	
	n	(%)	n	(%)	n	(%)	χ^2^	*p*
	339	(100.0)	114	(33.6)	225	(66.4)		
Age (years)								
18–24	88	(26.0)	28	(24.6)	60	(26.7)		ns
25–34	76	(22.4)	26	(22.8)	50	(22.2)		
35–44	69	(20.4)	18	(15.8)	51	(22.7)		
45–54	47	(13.9)	20	(17.5)	27	(12.0)		
55–65	35	(10.3)	12	(10.5)	23	(10.2)		
≥65	22	(6.5)	10	(8.8)	12	(5.3)		
BMI (kg/m^2^), categories								
<18.50 (underweight)	43	(12.7)	7	(6.1)	36	(16.0)	12.989	0.005
18.50–24.99 (normal weight)	179	(52.8)	62	(54.4)	117	(52.0)		
25.00–29.99 (overweight)	70	(20.7)	33	(28.9)	37	(16.4)		
≥30.00 (obese)	47	(13.9)	12	(10.5)	35	(15.6)		
Smoking								
No	131	(38.6)	38	(33.3)	93	(41.3)		ns
Yes	208	(61.4)	76	(66.7)	132	(58.7)		
Education								
None	164	(48.4)	52	(45.6)	112	(49.8)		ns
Some (1–8 yrs of school)	163	(48.1)	55	(48.2)	108	(48.0)		
Completed compulsory education	10	(2.9)	7	(6.1)	3	(1.3)		
Completed upper secondary school	2	(0.6)	0	(0.0)	2	(0.9)		
Employment type								
None	236	(69.8)	62	(54.9)	174	(77.3)	23.777	<0.0001
Occasional/Seasonal	88	(26.0)	41	(36.3)	47	(20.8)		
Permanent	14	(4.1)	10	(8.8)	4	(1.8)		
Hypertension								
No	218	(64.3)	75	(65.8)	131	(58.2)		ns
Yes	121	(35.7)	39	(34.2)	94	(41.8)		

**Table 3:** Univariate and multivariate logistic regression, odds of hypertension among Ukrainian Roma.

	Univariate			Multivariate		
	OR	95% CI	*p*-Value	OR	95% CI	*p*-Value
Gender						
Male	1.00	-	-	1.00	-	-
Female	1.38	0.86–2.21	0.178	1.90	1.07–3.38	0.029
Age, categorized						
18–24 years	1.00	-	-			
25–34 years	1.93	0.94–3.95	0.073			
35–44 years	2.09	1.01–4.33	0.048			
45–54 years	6.73	3.05–14.84	<0.0001			
55–65 years	9.11	3.75–22.16	<0.0001			
≥65 years	11.14	3.79–32.71	<0.0001			
Age, dichotomized						
<35 years	1.00	-	-			
≥35 years	3.41	2.15–5.41	<0.0001			
Age, dichotomized						
<45 years	1.00	-	-	1.00	-	-
≥45 years	5.21	3.16–8.57	<0.0001	6.14	3.50–10.75	<0.0001
BMI, categorized						
18.5–24.99 (normal)	1.00	-	-			
<18.5 (underweight)	0.53	0.23–1.22	0.134			
25.0–29.99 (overweight)	2.32	1.31–4.018	0.004			
>30.0 (obesity)	7.58	3.59–15.99	<0.0001			
BMI, dichotomized						
<25.0	1.00	-	-			
≥25.0	3.93	2.45–6.31	<0.0001			
BMI, dichotomized						
<30.0	1.00	-	-	1.00	-	-
≥30.0	6.51	3.18–13.35	<0.0001	8.92	3.95–20.13	<0.0001
Smoking						
No	1.00	-	-	1.00	-	-
Yes	2.18	1.36–3.48	0.001	3.20	1.81–5.69	<0.0001
Education, dichotomized						
None	1.00	-	-			
Some	0.79	0.51–1.23	0.300			
Years of education, dichotomized						
<9 years	1.00	-	-	1.00	-	-
≥9 years	0.53	0.24–1.16	0.113	0.50	0.20–1.25	ns
Employment status						
No	1.00	-	-	1.00	-	-
Yes	1.20	0.75–1.93	0.3407	1.50	0.85–2.64	ns
Employment type						
None	1.00	-	-			
Occasional/Seasonal	1.32	0.80–2.16	0.280			
Permanent	0.92	0.30–2.83	0.881			

## Data Availability

Data from the field study on Ukrainian Roma are available online: https://roma.inantro.hr/baza/ (accessed on 20 October 2020). In case of using this database for further analyses, please cite this publication. If further clarification is required, contact the corresponding authors.

## Supplementary Materials

The following are available online at www.mdpi.com/2075-4426/11/11/1138/s1, Figure S1: A flowchart of a selection process of studies eligible for the meta-analysis of prevalence of various CVD risk factors in the Roma population, Supplementary references—studies included in meta-analyses, Table S1: Distribution of cardiovascular risk factors in the Ukrainian Roma according to sex and age categories, Figure S2: Begg’s funnel plot of publications included in meta-analysis for smoking, Figure S3: Egger’s publication bias plot of publications included in meta-analysis for smoking, Figure S4: Influential meta-analysis plot with the effects estimates (ORs) for smoking after omitting an individual study each time, Figure S5: Begg’s funnel plot of publications included in meta–analysis for diabetes, Figure S6: Egger’s publication bias plot of publications included in meta-analysis for diabetes, Figure S7: Influential meta-analysis plot for adjusted effect estimates in odds ratio (OR) meta–analysis for diabetes, after omitting an individual study each time, Figure S8: Begg’s funnel plot of publications included in meta-analysis for abdominal obesity, Figure S9: Egger’s publication bias plot of publications included in meta-analysis for abdominal obesity, Figure S10: Influential meta–analysis plot for adjusted effect estimates in odds ratio (OR) meta–analysis for abdominal obesity, after omitting an individual study each time, Figure S11: Begg’s funnel plot of publications included in meta–analysis for metabolic syndrome, Figure S12: Egger’s publication bias plot of publications included in meta-analysis for metabolic syndrome, Figure S13: Influential meta–analysis plot for adjusted effect estimates in odds ratio (OR) meta–analysis for metabolic syndrome, after omitting an individual study each time, Figure S14: Begg’s funnel plot of publications included in meta–analysis for overweight and obesity (BMI ≥ 25 kg/m^2^), Figure S15: Egger’s publication bias plot of publications included in meta-analysis for overweight and obesity (BMI ≥ 25 kg/m^2^), Figure S16: Influential meta–analysis plot for adjusted effect estimates in odds ratio (OR) meta–analysis for overweight and obesity (BMI ≥ 25 kg/m^2^), after omitting an individual study each time, Figure S17: Begg’s funnel plot of publications included in meta–analysis for hypertension, Figure S18: Egger’s publication bias plot of publications included in meta-analysis for hypertension, Figure S19: Influential meta–analysis plot for adjusted effect estimates in odds ratio (OR) meta–analysis for hypertension, after omitting an individual study each time, Figure S20: Results of meta-analysis for obesity (BMI ≥ 30,00 kg/m^2^). The overall odds ratio was not statistically significant (*p* = 0.116), Table S2: A list of studies included in the supplementary meta-analyses, Table S3: Quality of studies included in the meta-analyses, Figure S21: Forest plot of the association between a representative sample of ethnic Roma (participants in the European project “Health and the Roma Community, Analysis of the Situation in Europe”—UNDP/WB/EC) and higher odds ratio (OR) for the prevalence of three CVD risk factors (obesity, smoking and diabetes), compared to a representative sample of the majority population (the Eurostat data). Results are stratified by country., Figure S22: Forest plot showing an association between a representative sample of ethnic Roma (participants in the European project “Health and the Roma Community, Analysis of the Situation in Europe”—UNDP/WB/EC) and lower odds ratio (OR < 1) for the prevalence of BMI ≥ 25.00 kg/m^2^ and hypertension, compared to a representative sample of the majority population (the Eurostat data). Results are stratified by country.
