# Supplementary material for: CVD Risk Factors in the Ukrainian Roma and Meta-Analysis of Their Prevalence in Roma Populations Worldwide
Source: J Pers Med. 2021 Nov 2;11(11):1138. doi: 10.3390/jpm11111138 (PMC8622536; doi:10.3390/jpm11111138)
Supplement: Supplementary file 1 [file jpm-11-01138-s001.zip › jpm-1404557-supplementary.pdf]

CVD risk factors in the Ukrainian Roma and meta-analysis of their prevalence in Roma populations worldwide

Matea Zajc Petranović[1*], Ashley E. Rizzieri[2], Dharshan Sivaraj[2,3], Nina Smolej Narančić[1], Tatjana Škarić-Jurić[1], Željka Celinšćak[1], Anita Stojanović Marković[1], Marijana Peričić Salihović[1], Julia Kalászi[2], Marianna Kalászi[2], John Q. Lin[3], Sanica Mehta[2], Jill Burleson[2], David A. Rizzieri[2*]

Affiliations:

[1] Institute for Anthropological Research, Gajeva 32, 10000 Zagreb, Croatia

[2] Division of Cellular Therapy, Duke University, 2400 Pratt Street, Durham, NC, 27708, USA

[3] Stanford University School of Medicine, 291 Campus Drive, Stanford, CA 94305, USA

***Correspondence:**

Matea Zajc Petranović
e-mail: matea@inantro.hr

David A. Rizzieri
e-mail: david.rizzieri@duke.edu

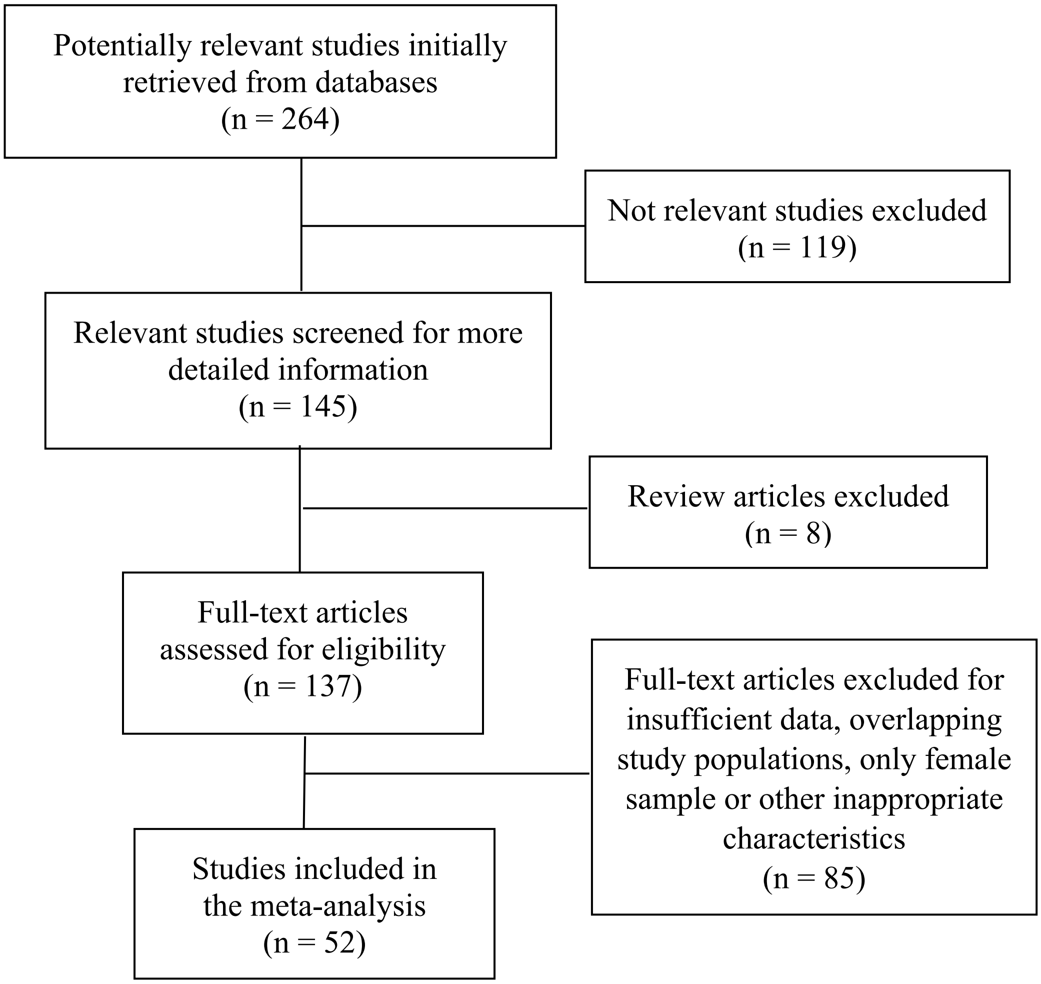

Figure S1. A flowchart of a selection process of studies eligible for the meta-analysis of prevalence of various CVD risk factors in the Roma population.

Supplementary references – studies included in meta-analyses

1. Adámková, V.; *et al*. Genetic and biochemical characteristics in the Roma minority in the South Bohemia Region. *Neuro. Endocrinol. Lett.* **2015**, *36(Suppl.2)*, 29–34.
2. AITHS. All Ireland Traveller Health Study – Our Geels. *Sch Public Heal Physiother Popul Sci*. **2010**, Available online: https://www.ucd.ie/t4cms/AITHS_SUMMARY.pdf (accessed on September 4th 2021).
3. Alves, L.; Azevedo, A.; Barros, H.; Vollenweider, P.; Waeber, G.; Marques-Vidal, P. Prevalence and management of cardiovascular risk factors in Portuguese living in Portugal and Portuguese who migrated to Switzerland. *BMC Public Health.* **2015**, *15*, 307; DOI:10.1186/s12889-015-1659-8.
4. Aranceta-Bartrina, J.; Pérez-Rodrigo, C.; Alberdi-Aresti, G.; Ramos-Carrera, N.; Lázaro-Masedo, S. Prevalence of General Obesity and Abdominal Obesity in the Spanish Adult Population (Aged 25–64 Years) 2014–2015: The ENPE Study. *Rev. Española. Cardiol.* English Ed. **2016**, *69(6)*, 579–587.
5. Babinska, I. *et al*. Is the cardiovascular risk profile of people living in Roma settlements worse in comparison with the majority population in Slovakia? *Int. J. Public Health.* **2013**, *58(3)*, 417–425.
6. Balanda, K. P. *et al.* Prevalence of Diabetes in the Republic of Ireland: Results from the National Health Survey (SLAN) 2007. *PLoS One.* **2013**, *8(10):e78406*; DOI:10.1371/journal.pone.0078406.
7. Barron; S.; Balanda, K.; Hughes, J.; Fahy, L. National and subnational hypertension prevalence estimates for the Republic of Ireland: Better outcome and risk factor data are needed to produce better prevalence estimates. *BMC Public Health.* **2014**, *14(1)*, 1–10.
8. Bartos, D. *et al*. The prevalence of major cardiovascular risk factors in a Roma ethnic sample population. *Eur. Heart. J.* **2013**, *34(Suppl.1)*, 2540.
9. Beljić Živković, T. *et al*. Screening for diabetes among Roma people living in Serbia. *Croat. Med. J.* **2010**, *51(2)*, 144-150.
10. Bilovol, O.; Shaposhnikova, Y.; Ilchenko, I.; Shalimova, A. Relationship between peculiarities of atrial fibrillation, body mass index and adipokines levels. *Vessel Plus.* **2017**, *1*, 196–201.
11. Borissova, A.-M. I.; Shinkov, A. D.; Vlahov, J. D.; Dakovska, L. N.; Todorov, T.C. Prevalence of arterial hypertension in Bulgarian population (actual data). *Endokrinologya.* **2015a***, 20(2)*, 76-81.

12. Borissova, A.-M. I.; Shinkov, A. D.; Vlahov, J. D.; Dakovska, L. N.; Todorov, T.C. Survey on the prevalence of obesity in Bulgarian population in 2012 year. *Endokrinologya*. **2015b**, *20(2)*, 82-88.

13. Chagarna, N. S.; Andreeva, T.I. Lifestyle correlates of overweight and obesity among the population of Ukraine. *Tobacco Control and Public Health in Eastern Europe*. **2014**, *4(1)*, 15-28; DOI:10.6084/m9.figshare.1060345.

14. Donfrancesco, C. *et al*. Italian network for obesity and cardiovascular disease surveillance: A pilot project. *BMC Fam. Pract.* **2008**, *9*, 53; DOI:10.1186/1471-2296-9-53.

15. Enache, G.; Rusu, E.; Ilinca, A.; Rusu, F.; Costache, A.; Radulian, G. Prevalence of obesity and newly diagnosed diabetes in the Roma population from a county in the south part of Romania (Călăraşi county) - Preliminary results. *Rom. J.Diabetes Nutr. Metab. Dis.* **2016**, *23(1)*, 27–36; DOI:10.1515/rjdnmd-2016-0004.

16. Erceg, M.; Kern, J.; Babić-Erceg, A.; Ivičević-Uhernik, A.; Vuletić, S. Regional differences in the Prevalence of arterial hypertension in Croatia. *Coll. Antropol.* **2009**, *33(Suppl.1)*, 19–23.

17. Fedačko, J. *et al*. Prevalence of cardiovascular risk factors in relation to metabolic syndrome in the Roma population compared with the non-Roma population in the Eastern part of Slovakia. *Cent. Eur. J. Public Health.* **2014**, *22(Cvd)*, S69–S74.

18. Fundación Secretariado Gitano. Health and the Roma Community, analysis of the situation in Europe. Bulgaria, Czech Republic, Greece, Portugal, Romania, Slovakia, Spain. FSG. 2009. Available online: http://romani.humanities.manchester.ac.uk/virtuallibrary/librarydb/web/files/pdfs/155/VL-070.pdf (accessed on September 13th 2021).

19. Gikas, A.; Lambadiari, V.; Sotiropoulos, A.; Panagiotakos, D.; Pappas, S. Prevalence of Major Cardiovascular Risk Factors and Coronary Heart Disease in a Sample of Greek Adults: The Saronikos Study. *Open Cardiovasc. Med. J.* **2016**, *10(1)*, 69–80.

20. Gualdi-Russo, E.; Zironi, A.; Dallari, G. V.; Toselli, S. Migration and health in Italy: A multiethnic adult sample. *J. Travel. Med.* **2009**, *16(2)*, 88–95.

21. Hadjiev, D.I.; Mineva, P. P.; Vukov, M.I. Multiple modifiable risk factors for first ischemic stroke: A population-based epidemiological study. *Eur. J. Neurol.* **2003**, *10(5),* 577–582.

22. Healthy Ireland Survey – Summary of findings 2015. Available online: https://assets.gov.ie/16210/525a06d3aaef4f23889c8fbdcc40d40a.pdf (accessed on September 13th 2021).

23. Hidvegi, T.; Hetyesi, K.; Biro, L.; Nadas, J.; Jermendy, G. Screening for metabolic syndrome within a minority ethnic group (adult Gypsy people) in Hungary. *Bratisl. Lek. List.* **2012**, *113(12)*, 721–724.

24. Ivičević Uhernik, A.; Erceg, M. Prevalence of arterial hypertension – Croatian Health Survey 2008. Book of abstracts 2nd Croatian Congress On Preventive Medicine and Health Promotion with internationa participation, Zagreb, Croatia, October 13 – 16 2010; Selma Šogorić, Danijela Štimac; Zagreb, Croatia, 2010; DIZ -272, 33.

25. Janković, J.; Davidović, M.; Bjegović-Mikanović, V.; Janković, S. Status of cardiovascular health in the Republic of Serbia: Results from the National health Survey. *PLoS One.* **2019**, *14(3)*:*e0214505*; DOI:10.1371/journal.pone.0214505.

26. Jiménez-Sánchez, S. *et al*. Prevalence of Migraine Headaches in the Romany Population in Spain: Sociodemographic Factors, Lifestyle and Co-Morbidity. *J. Transcult. Nurs.* **2013**, *24(1)*, 6–13.

27. Kósa, Z. *et al*. Prevalence of metabolic syndrome among Roma: A comparative health examination survey in Hungary. *Eur. J. Public Health.* **2014**, *25(2)*, 299–304.

28. Krajcovicova-Kudlackova, M.; Blazicek, P.; Ginter, E.; Spustova, V. Insulin levels in Gipsy minority. *Bratisl. Lek. Listy.* **2002**, *103(12)*, 459–461.

29. Krajcovicova-Kudlackova, M.; Blazicek, P.; Spustova, V.; Valachovicova, M.; Ginter, E. Cardiovascular risk factors in young Gypsy population. *Bratisl. Lek. Listy.* **2004**, *105(7-8)*, 256-259.

30. Maksimović, M.; Gudelj Rakić, J.M.; Vlajinac, H.D.; Vasiljević, N.D.; Nikić, M.I.; Marinković, J.M. Comparison of different anthropometric measures in the adult population in Serbia as indicators of obesity: Data from the National Health Survey 2013. *Public Health Nutr.* **2016**, *19(12)*, 2246-2255.

31. Metelko, Ž.; Pavlić-Renar, I.; Poljičanin, T.; Szirovitza, L.; Turek, S. Prevalence of diabetes mellitus in Croatia. *Diabetes Res. Clin. Pract.* **2008**, *81(2)*, 263-267.

32. Music Milanović, S. Demografske, bihevioralne i socioekonomske odrednice debljine u odraslih u Hrvatskoj. PhD Thesis, University of Zagreb – School of Medicine, Zagreb, Croatia, 2010. Available online: http://medlib.mef.hr/932/1/Music-Milanovic_S_disertacija_rep_932.pdf (accessed on October 13th 2020)

33. Nagy, K.; Fiatal, S.; Sándor, J.; Ádány, R. Distinct penetrance of obesity-associated susceptibility alleles in the Hungarian general and Roma populations. *Obes. Facts.* **2017**, *10(5)*, 444-457.

34. Naydenov, S.; Torbova, S.; Nikolov, F.; Tsonev, S.; Naydenova, N. Abdominal obesity, blood pressure and diabetes mellitus in Bulgarian urban population. *J. Hypertens.* **2011**, *29:* e432.

35. ObEpi. Enquête épidémiologique nationale sur le surpoids et l'obésité. 2012; Available online: http://www.roche.fr/content/dam/corporate/roche_fr/doc/obepi_2012.pdf (accessed on September 13th 2021).

36. Papon, C.; Delarche, N.; Le Borgne, C.; Bauduer, F. Assessment of cardiovascular risk factors in a Roma community from Southwestern France. *Am. J. Hum. Biol.* **2017**, *29(1)*, 1-5.

37. Parry, G.; Van Cleemput, P.; Peters, J.; Walters, S.; Thomas, K. & Cooper, C. Health status of Gypsies and Travellers in England. *J. Epidemiol. Community Health.* **2007**, *61(3)*, 198-204.

38. Paulik, E.; Nagymajtényi, L.; Easterling, D.; Rogers, T. Smoking behaviour and attitudes of Hungarian Roma and non-Roma population towards tobacco control policies. *Int. J. Public Health.* **2011**, *56(5)*, 485-491.

39. Pavlovski, B. Health, Health Care, and Impact on the Health of the Roma in the Republic of Macedonia. Assoc Emancip Solidar Equal Women Repub Maced. Published by Association for Emancipation, Solidarity and Equality of Women of Republic of Macedonia. Available online: http://www.esem.org.mk/en/pdf/Publikacii/Ostanati/2008/Health,%20health%20care%20and%20impacts%20on%20the%20health%20of%20the%20Roma%20in%20RM_Final%20version.pdf (accessed on September 13th 2021).

40. Petrikova, J. *et al*. Serum uric acid in Roma and non-Roma—its correlation with metabolic syndrome and other variables. *Int. J. Environ. Res. Public Health.* **2018**, *15(7),* 1412; DOI:10.3390/ijerph15071412.

41. Poveda, A.; Ibáñez, M. E.; Rebato, E. Obesity and body size perceptions in a Spanish Roma population. *Ann. Hum. Biol.* **2014**, *41(5)*, 428–435.

42. Pradhan, D. The association between smoking and hypertension in Ukraine. Master`s thesis in Public Health, University of Eastern Finland - School of Medicine Faculty of Health Sciences, Kuopio, Finland, January 2014. Available online: https://epublications.uef.fi/pub/urn_nbn_fi_uef-20140570/index_en.html (accessed on September 13th 2021).

43. Rexhepi, A.; Elezi, N.; Jani, Y. Prevalence of Metabolic Syndrome in Adult Population of District of Tetovo in the Northwest Region of the Republic of Macedonia. *Int. J. Med. Res. Heal. Sci.* **2018**, *7(3)*, 104–114.

44. Samardžić, S.; Vuletić, G. Prevalence of smoking in Croatia – Croatian Health Survey 2008. Book of abstracts 2nd Croatian Congress On Preventive Medicine and Health Promotion with internationa participation, Zagreb, Croatia, October 13 – 16 2010; Selma Šogorić, Danijela Štimac; Zagreb, Croatia, 2010; JZI -183, 36.

45. Slattery, D.; Brennan, M.; Canny, C.; Avalos, G.; Dunne, F. The Prevalence of Diabetes, Pre Diabetes and the Metabolic Syndrome in Irish Travellers. American Diabetes Association 71st Scientific Session, San Diego, California, United States of America, June 24-28 2011; 2011; Abstract 2505-PO.

46. Sudzinova, A. *et al*. Seven years' mortality in Roma and non-Roma patients after coronary angiography. *Eur. J. Public Health.* **2015**, *25(5)*, 765–769.

47. Šedova, L. *et al*. Evaluation of selected indicators of overweight and obesity of Roma minority in the region of South Bohemia. *Neuro. Endocrinol. Lett.* **2015**, *36(S2)*, 35-42.

48. Urban, D.; Kajanová, A. Smoking and alcohol consumption in Romany communities in the Czech and Slovak Republics. *Kontakt.* **2011**, *13(3)*, 328–335.

49. Vanova, A.; Skyvova, M.; Maly, M. The use of tobacco in the Czech Republic. National Public Health Institute 2018, Available online: http://www.szu.cz/uploads/documents/czzp/zavislosti/the_use_of_tobacco_in_the_czech_republic_2017.pdf (accessed on September 13th 2021).

50. Vozarova De Courten, B.; *et al*. Higher prevalence of type 2 diabetes, metabolic syndrome and cardiovascular diseases in Gypsies than in non-Gypsies in Slovakia. *Diabetes Res. Clin. Pract.* **2003**, *62(2)*, 95–103.

51. Waterhouse, D.F.; McLaughlin, A.M.; Sheehan, F.; O'Shea, D. An examination of the prevalence of IDF- and ATPIII-defined metabolic syndrome in an Irish screening population. *Ir. J. Med. Sci.* **2009**, *178(2)*, 161-166.

52. Zeljko, H.M.; *et al*. Age trends in prevalence of cardiovascular risk factors in Roma minority population of Croatia. *Econ. Hum. Biol.* **2013**, *11(3)*, 326–336.

	N (%)	M/F (%/%)	18-24 yrs N (%)	25-34 yrs N (%)	35-44 yrs N (%)	45-54 yrs N(%)	55-64 yrs N (%)	≥65 yrs N (%)	p
Overweight and obese (BMI≥25 kg/m^2)	117 (34.5)	45/72 (39.5/32.0)	12 (13.6)	26 (34.2)	27 (39.1)	25 (53.2)	19 (54.3)	8 (36.4)	<0.0001
Obese (BMI≥30 kg/m^2)	47 (13.9)	12/35 (10.5/15.6)	6 (6.8)	9 (11.8)	12 (17.4)	7 (14.9)	8 (22.9)	5 (22.7)	ns
Hypertension (BP≥140/90 mmHg)	133 (39.2)	39/94 (34.2/41.8)	17 (19.3)	24 (31.6)	23 (33.3)	29 (61.7)	24 (68.6)	16 (72.7)	<0.0001
Smoking	208 (61.4)	76/132 (66.7/58.7)	39 (44.3)	50 (65.8)	51 (73.9)	32 (68.1)	23 (65.7)	13 (59.1)	<0.01

Table S1. Distribution of cardiovascular risk factors in the Ukrainian Roma according to sex and age categories.

Supplementary material – results of tests for publication bias (Begg`s and Egger`s) in meta-analyses of the investigated CVD risk factors, and meta-influential analyses

<u>Smoking</u>

Tests for Publication Bias

Begg's Test

adj. Kendall's Score (P-Q) = -8
Std. Dev. of Score = 16.39
Number of Studies = 13
z = -0.49
Pr > |z| = 0.625
z = 0.43 (continuity corrected)
Pr > |z| = 0.669 (continuity corrected)

Egger's test

Std_Eff	Coef.	Std. Err.	t	P>|t|	[95% Conf. Interval]	
slope	1.602309	0.4352129	3.68	0.004	0.6444116	2.560206
bias	-4.290866	4.325193	-0.99	0.342	-13.81055	5.22882

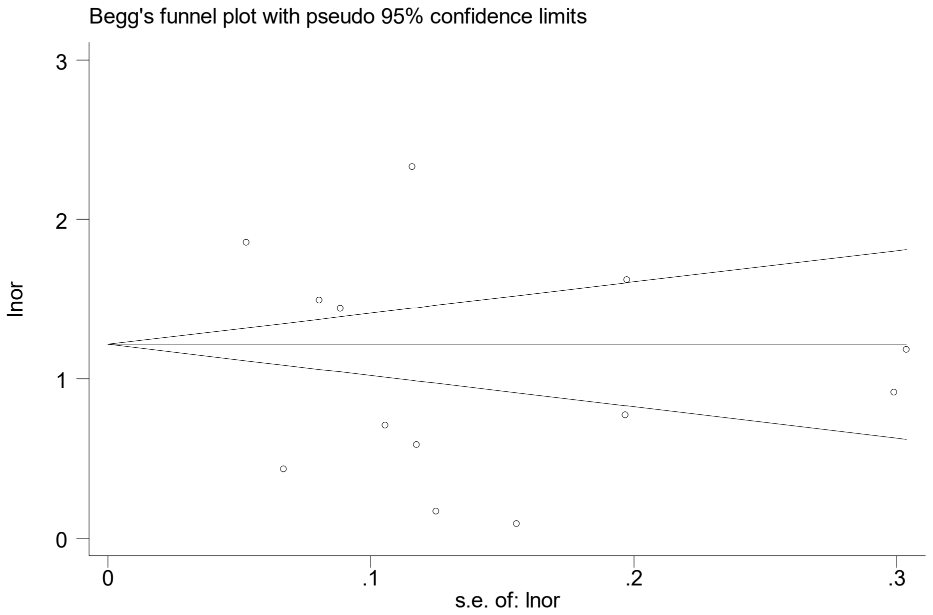

Figure S2. Begg`s funnel plot of publications included in meta-analysis for smoking.

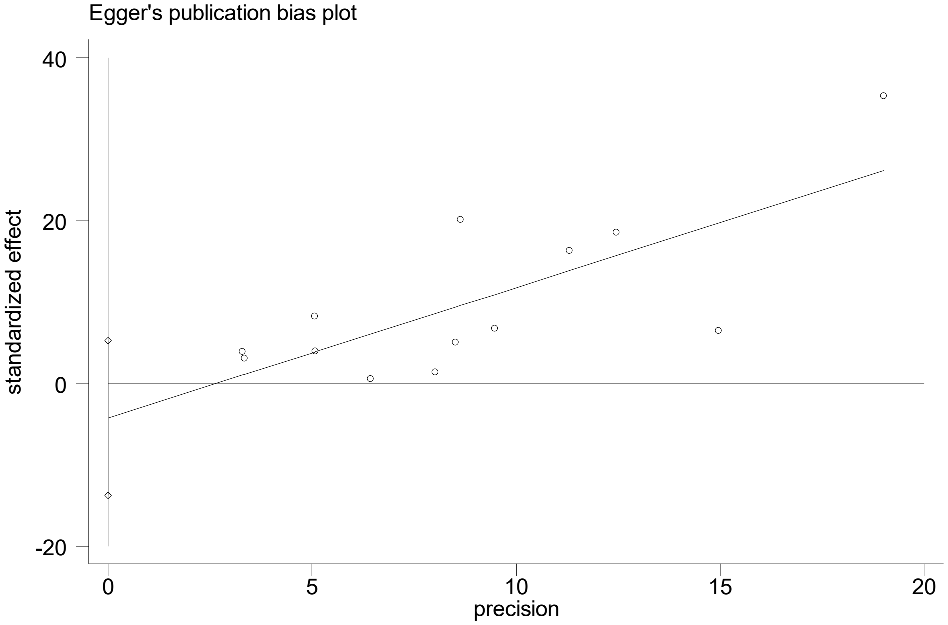

Figure S3. Egger`s publication bias plot of publications included in meta-analysis for smoking.

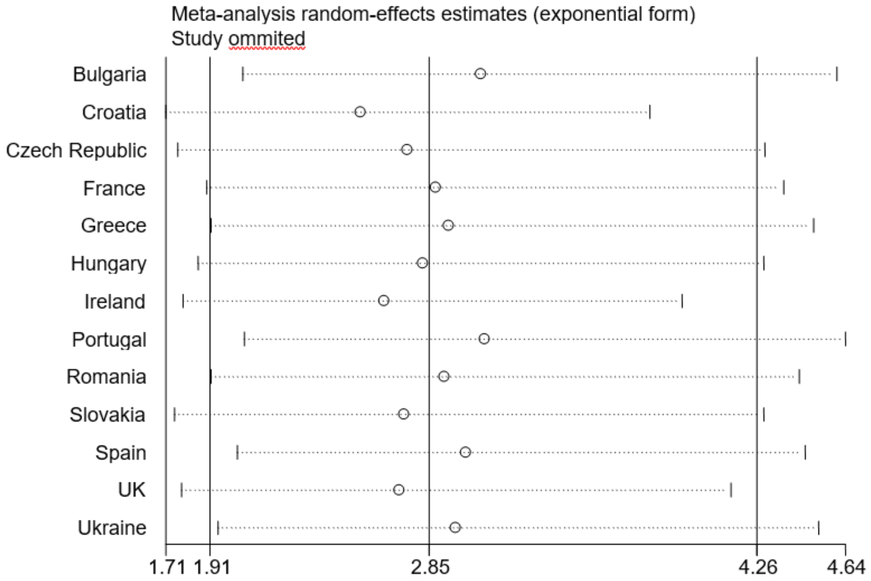

Figure S4. Influential meta-analysis plot with the effects estimates (ORs) for smoking after omitting an individual study each time.

Diabetes

Tests for Publication Bias

Begg's Test

```
  adj. Kendall's Score (P-Q) =      8
          Std. Dev. of Score =  16.39
           Number of Studies =     13
                          z  =   0.49
                    Pr > |z| =  0.625
                          z  =   0.43 (continuity corrected)
                    Pr > |z| =  0.669 (continuity corrected)
```

Egger's test

Std_Eff	Coef.	Std. Err.	t	P>|t|	[95% Conf. Interval]	
slope	0.0664472	0.3142009	0.21	0.836	-0.6251043	0.7579987
bias	1.364926	1.982896	0.69	0.505	-2.999398	5.72925

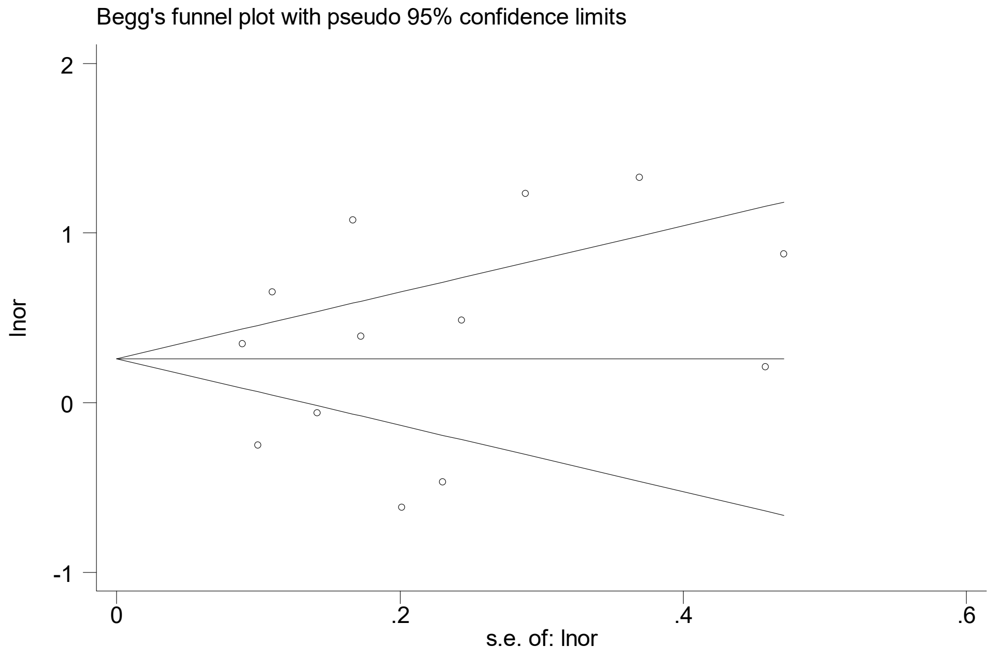

Figure S5. Begg`s funnel plot of publications included in meta–analysis for diabetes.

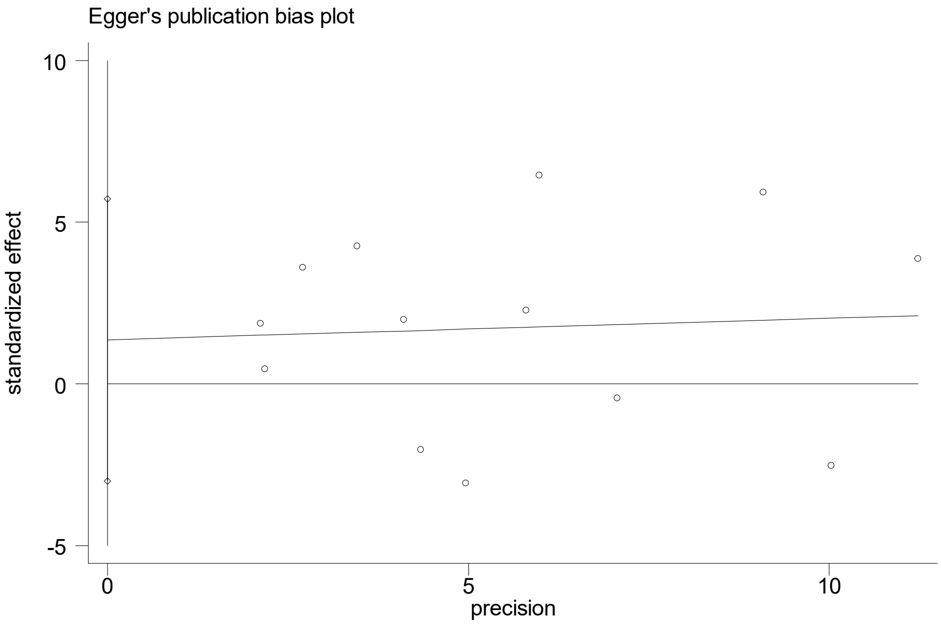

Figure S6. Egger`s publication bias plot of publications included in meta-analysis for diabetes.

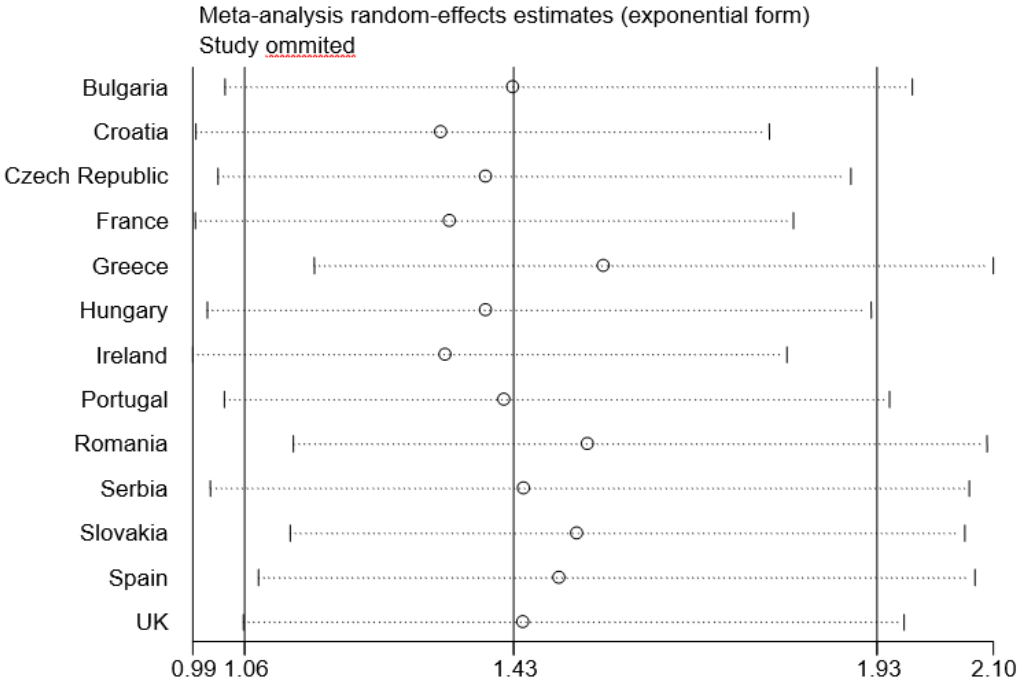

Figure S7. Influential meta-analysis plot for adjusted effect estimates in odds ratio (OR) meta–analysis for diabetes, after omitting an individual study each time.

Abdominal obesity

Tests for Publication Bias

Begg's Test

```
  adj. Kendall's Score (P-Q) =        7
          Std. Dev. of Score =    11.18
           Number of Studies =       10
                         z  =     0.63
                  Pr > |z| =    0.531
                         z  =     0.54 (continuity corrected)
                  Pr > |z| =    0.592 (continuity corrected)
```

Egger's test

```
------------------------------------------------------------------------------
     Std_Eff |      Coef.   Std. Err.      t    P>|t|     [95% Conf. Interval]
-------------+----------------------------------------------------------------
       slope |  -.0627253    0.528452    -0.12   0.908    -1.281338    1.155887
        bias |   2.047115    4.712348     0.43   0.675    -8.819578    12.91381
```

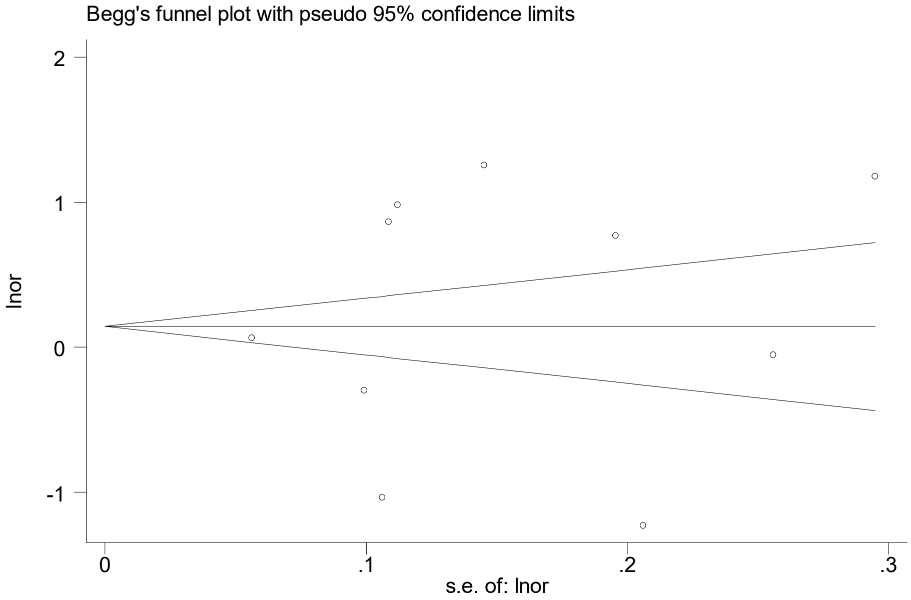

Figure S8. Begg`s funnel plot of publications included in meta-analysis for abdominal obesity.

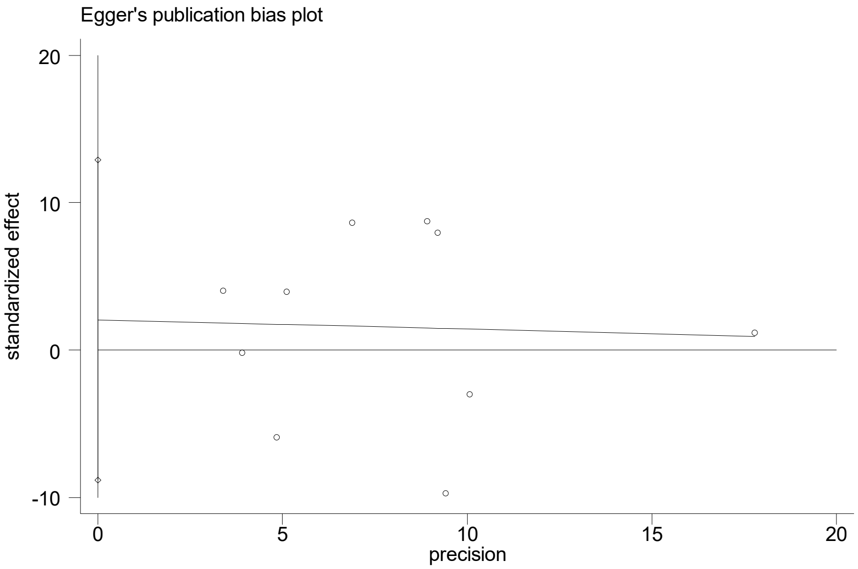

Figure S9. Egger`s publication bias plot of publications included in meta-analysis for abdominal obesity.

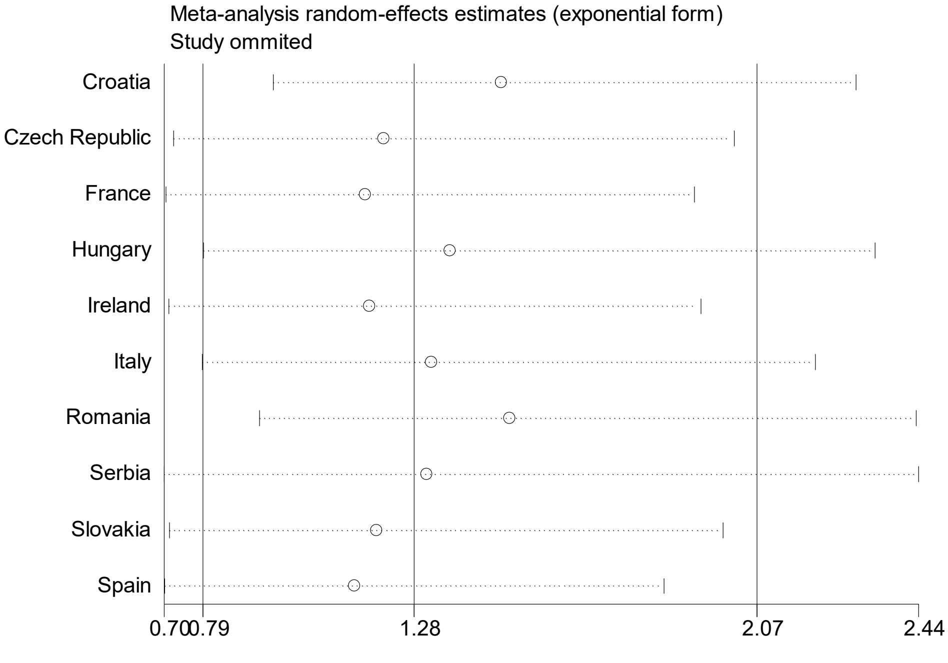

Figure S10. Influential meta–analysis plot for adjusted effect estimates in odds ratio (OR) meta–analysis for abdominal obesity, after omitting an individual study each time.

Metabolic Syndrome

Tests for Publication Bias

Begg's Test

```
  adj. Kendall's Score (P-Q) =       3
          Std. Dev. of Score =    1.91
           Number of Studies =       3
                         z  =    1.57
                    Pr > |z| =   0.117
                         z  =    1.04 (continuity corrected)
                    Pr > |z| =   0.296 (continuity corrected)
```

Egger's test

```
------------------------------------------------------------------------------
    Std_Eff |      Coef.   Std. Err.      t    P>|t|     [95% Conf. Interval]
-------------+----------------------------------------------------------------
      slope |  -1.840566   0.3009939    -6.11   0.103    -5.665056    1.983924
       bias |   21.22612   2.639877     8.04   0.079     -12.3167    54.76894
```

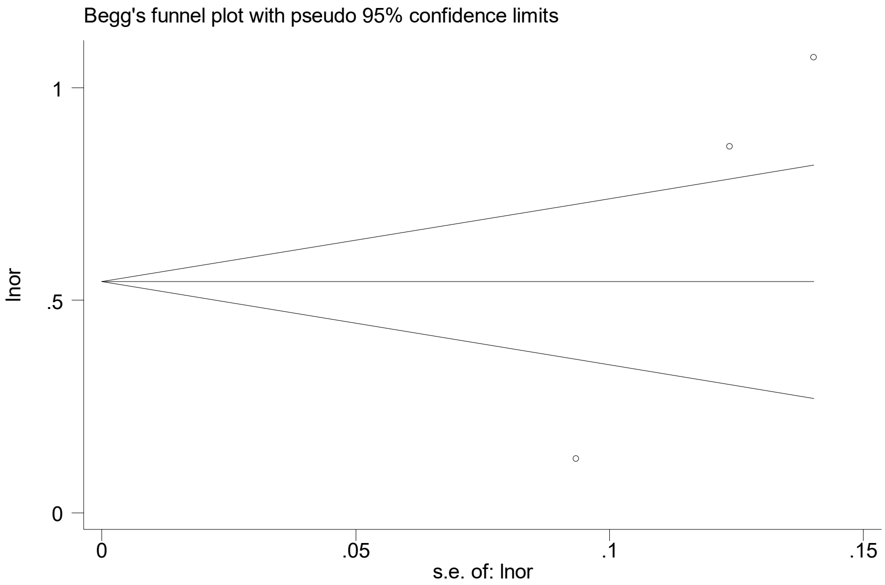

Figure S11. Begg`s funnel plot of publications included in meta–analysis for metabolic syndrome.

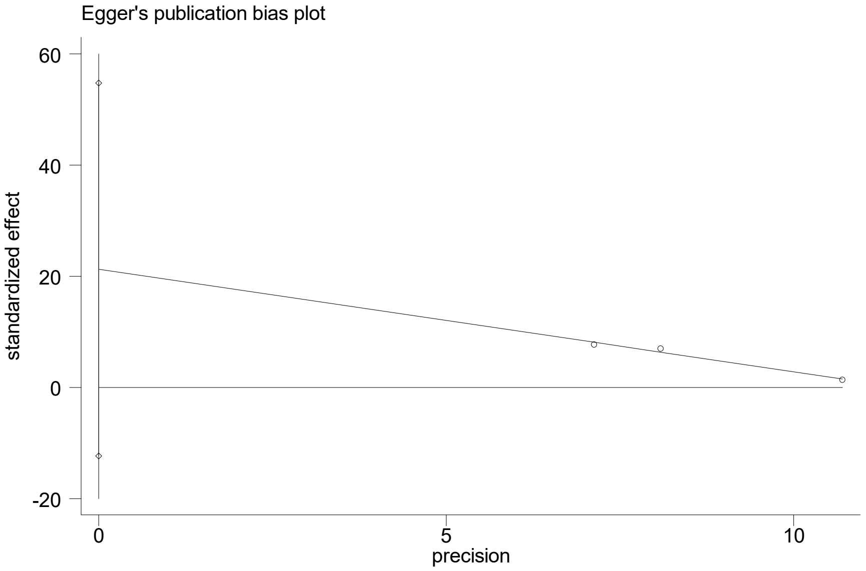

Figure S12. Egger`s publication bias plot of publications included in meta-analysis for metabolic syndrome.

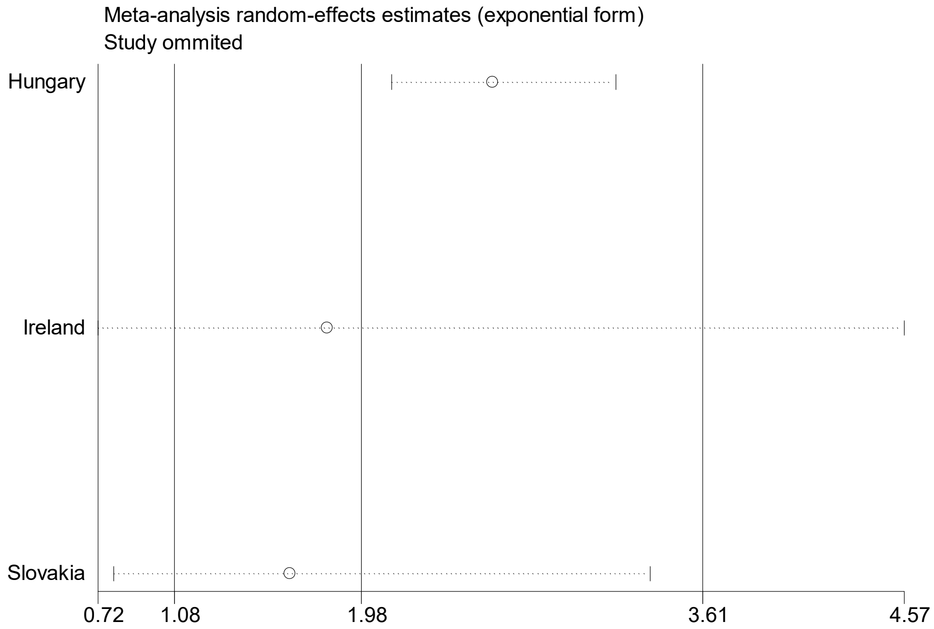

Figure S13. Influential meta–analysis plot for adjusted effect estimates in odds ratio (OR) meta–analysis for metabolic syndrome, after omitting an individual study each time.

Overweight and obesity (BMI≥25kg/m²)

Tests for Publication Bias

Begg's Test

```
  adj. Kendall's Score (P-Q) =       5
         Std. Dev. of Score =   11.18
          Number of Studies =      10
                         z  =    0.45
                   Pr > |z| =   0.655
                         z  =    0.36 (continuity corrected)
                   Pr > |z| =   0.721 (continuity corrected)
```

Egger's test

```
------------------------------------------------------------------------------
    Std_Eff |      Coef.   Std. Err.      t    P>|t|     [95% Conf. Interval]
-------------+----------------------------------------------------------------
      slope |  -0.4733091   1.130636    -0.42   0.687     -3.08056    2.133942
       bias |   1.856511   8.324784     0.22   0.829    -17.34048     21.0535
------------------------------------------------------------------------------
```

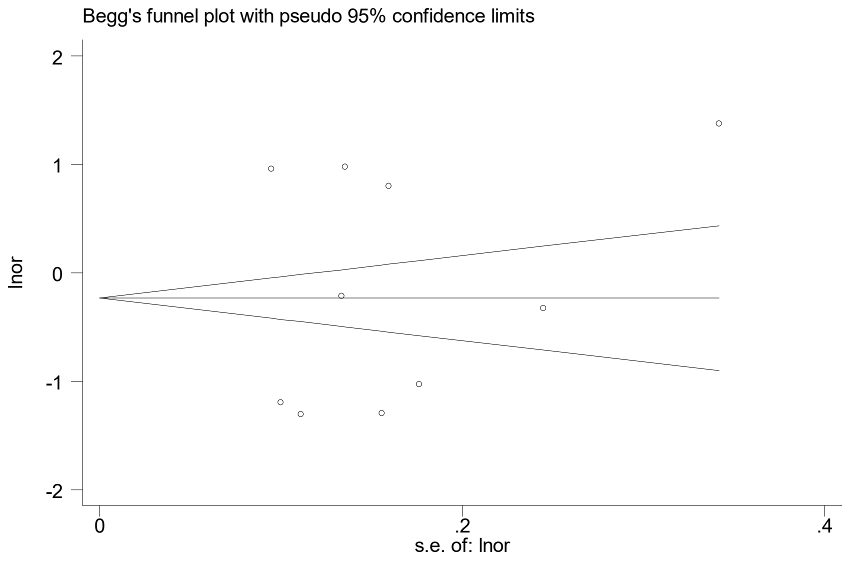

Figure S14. Begg`s funnel plot of publications included in meta–analysis for overweight and obesity (BMI≥25 kg/m²).

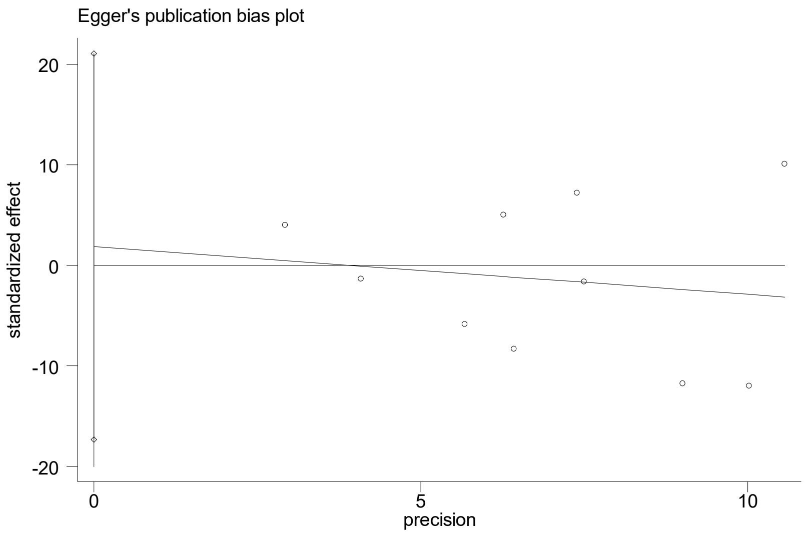

Figure S15. Egger`s publication bias plot of publications included in meta-analysis for overweight and obesity (BMI≥25 kg/m^2).

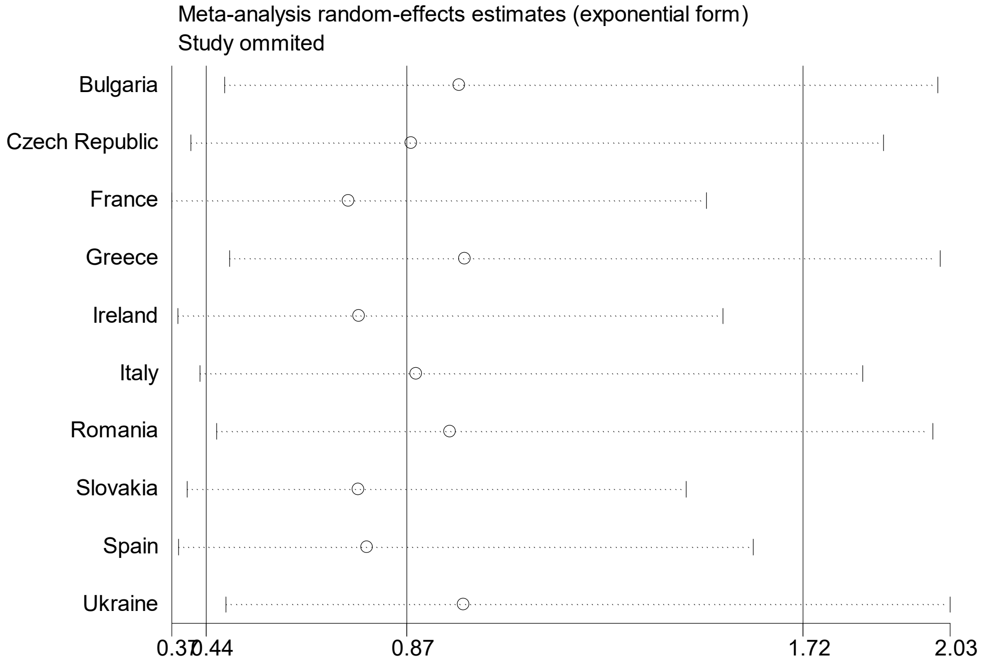

Figure S16. Influential meta–analysis plot for adjusted effect estimates in odds ratio (OR) meta–analysis for overweight and obesity (BMI≥25 kg/m^2), after omitting an individual study each time.

Hypertension

Tests for Publication Bias

Begg's Test

adj. Kendall's Score (P-Q) = -5
Std. Dev. of Score = 18.27
Number of Studies = 14
z = -0.27
Pr > |z| = 0.784
z = 0.22 (continuity corrected)
Pr > |z| = 0.827 (continuity corrected)

Egger's test

Std_Eff	Coef.	Std. Err.	t	P>|t|	[95% Conf. Interval]	
slope	0.4785389	0.4907615	0.98	0.349	-0.5907384	1.547816
bias	0.1623843	4.117593	0.40	0.969	-8.809081	9.133849

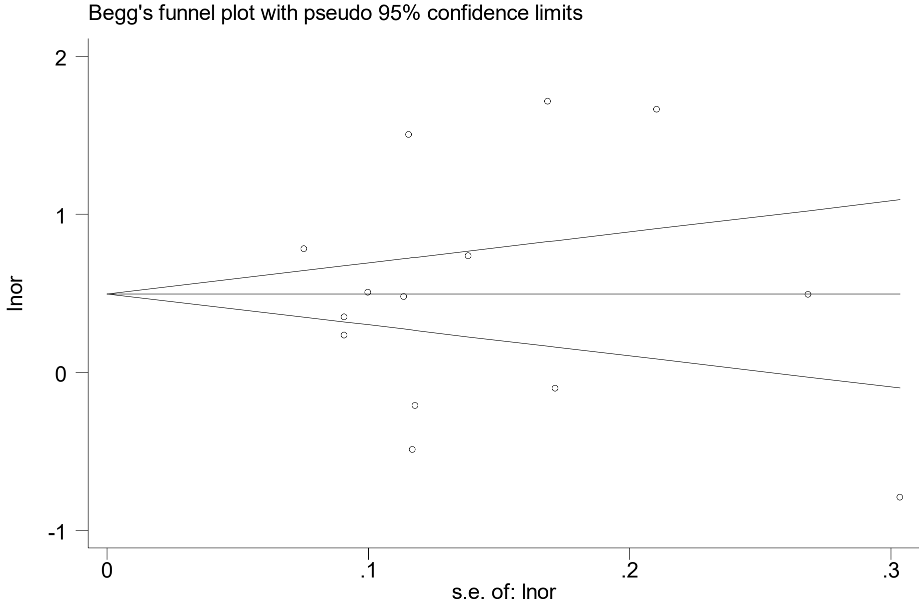

Figure S17. Begg`s funnel plot of publications included in meta–analysis for hypertension.

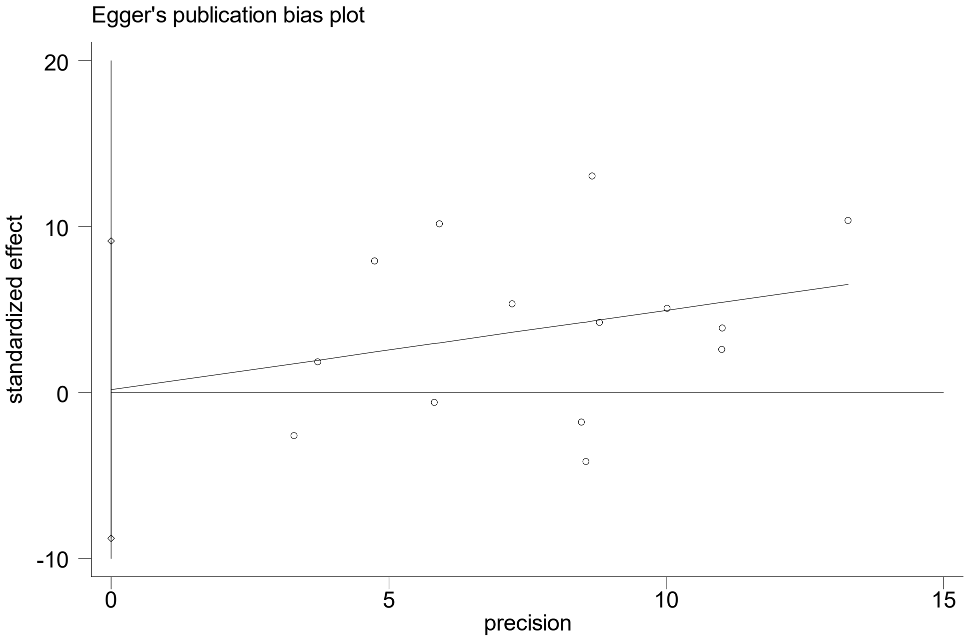

Figure S18. Egger`s publication bias plot of publications included in meta-analysis for hypertension.

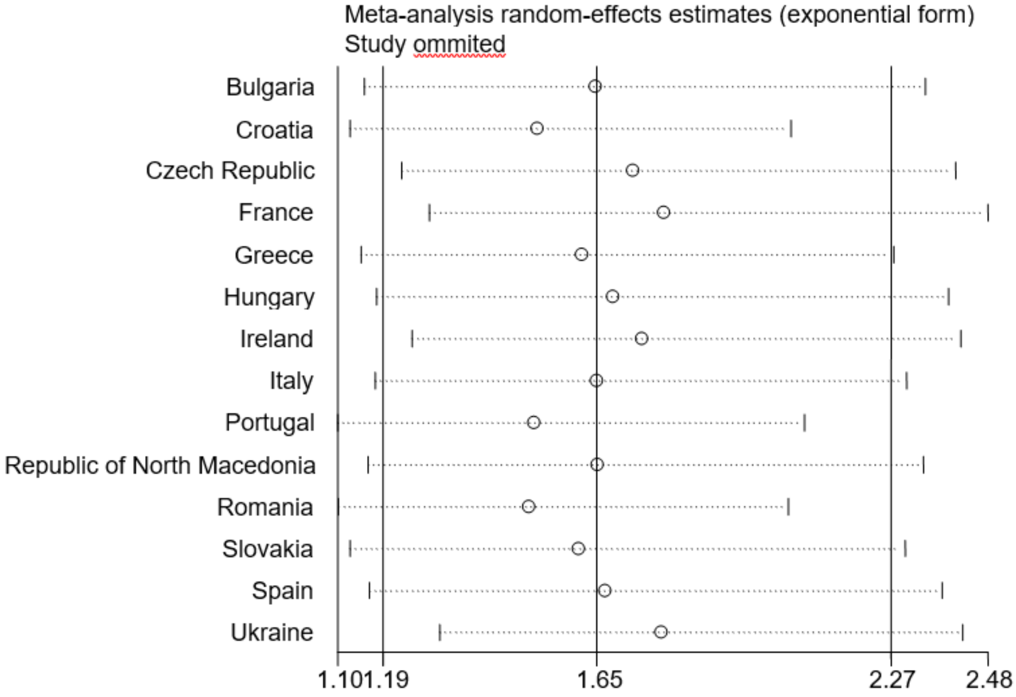

Figure S19. Influential meta–analysis plot for adjusted effect estimates in odds ratio (OR) meta–analysis for hypertension, after omitting an individual study each time.

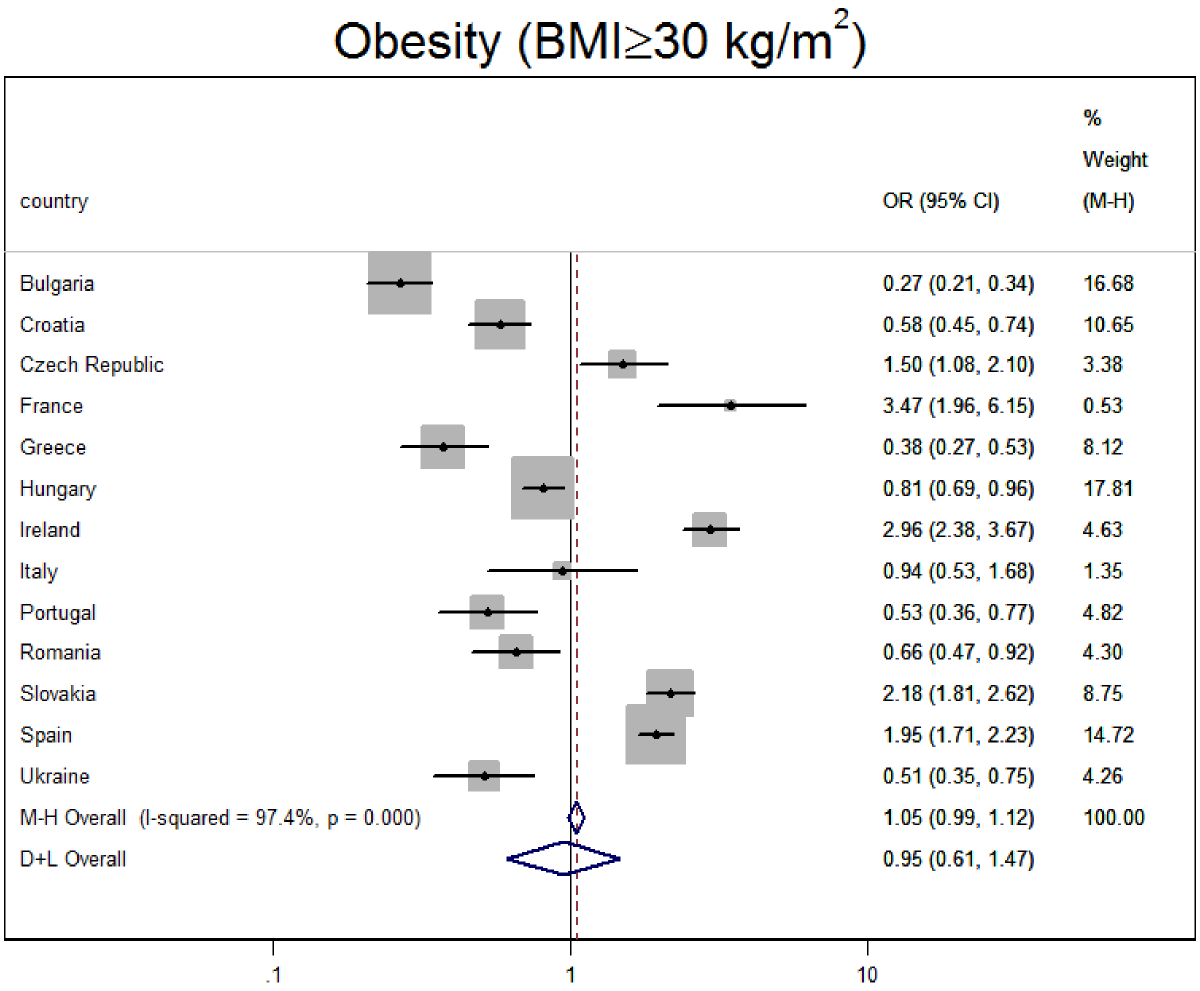

Figure S20. Results of meta-analysis for obesity (BMI≥30,00 kg/m^2). The overall odds ratio was not statistically significant (p=0.116).

Table S2. A list of studies included in the supplementary meta-analyses.

Roma population

Country	Diabetes mellitus	Hypertension	Smoking	N	Overweight & obese	Obese	N_over_ob
Bulgaria	9.3	32.5	46.1	548	41.7	14.2	786
Czech Republic	8.5	17.2	58.4	681	47.9	20.2	984
Greece	6.7	16.3	56.3	426	32.7	9.4	629
Romania	6.9	17.5		1592	43.4	16.9	2554
Slovakia	6.0	17.7	53.2	336	43.7	17.9	639
Spain	5.7	14.9	34.1	992	49.2	19.7	1446
total				4575			*7388

EHIS 1 wave

Country	Diabetes mellitus	Hypertension	Smoking	Overweight & obese	Obese	Sample size
Bulgaria	4.2	26.5	29.2	50.8	11.5	5661
Czech Republic	6.1	26.4	24.3	56.6	18.3	1955
Greece	7.7	21.4	31.8	56.3	17.6	6172
Romania	3.1	16.5	20.5	50.3	7.9	18172
Slovakia	6	29.7	19.3	50.7	15.1	4972
Spain	5.9	19.3	25.2	53	15.7	22188

*both minors and adults

The prevalence of CVD risk factors in Roma populations and sample sizes were found in the following paper: Fundación Secretariado Gitano. Health and the Roma Community, analysis of the situation in Europe. Bulgaria, Czech Republic, Greece, Portugal, Romania, Slovakia, Spain. FSG. 2009.

The prevalence of hypertension and diabetes mellitus was taken from the Eurostat EHIS folder Health status – historical data (2008), while the prevalence of nutritional status categories and smoking from the EHIS folder Health determinants – historical status (2008).

Table S3. Quality of studies included in the meta-analyses.

	YES		NO		NA*	
	COUNT	%	COUNT	%	COUNT	%
Was the study design appropriate?	51	100	0	0	0	0
Was the research question or objective in this paper clearly stated?	46	90.2	4	7.9	1	1.9
Was the study population clearly specified and defined?	48	94.1	3	5.9	0	0
Was there a matching majority population from the same geographic area for each Roma population included in meta-analyses?	51	100	0	0	0	0
Were inclusion and exclusion criteria for being in the study prespecified and applied uniformly to all participants?	51	100	0	0	0	0
Were a sample size justification, power description, or variance and effect estimates provided?	48	94.1	2	3.9	1	1.9
Were the outcome measures (dependent variables) clearly defined, valid, reliable, and implemented consistently across all study participants?	43	84.3	8	15.7	0	0
Was the conflict of interest stated?	49	96.1	0	0	2	3.9
Were titles, abstracts, and full-text articles dually and independently reviewed for inclusion and exclusion to minimize bias?	51	100	0	0	0	0

*NOT APPLICABLE

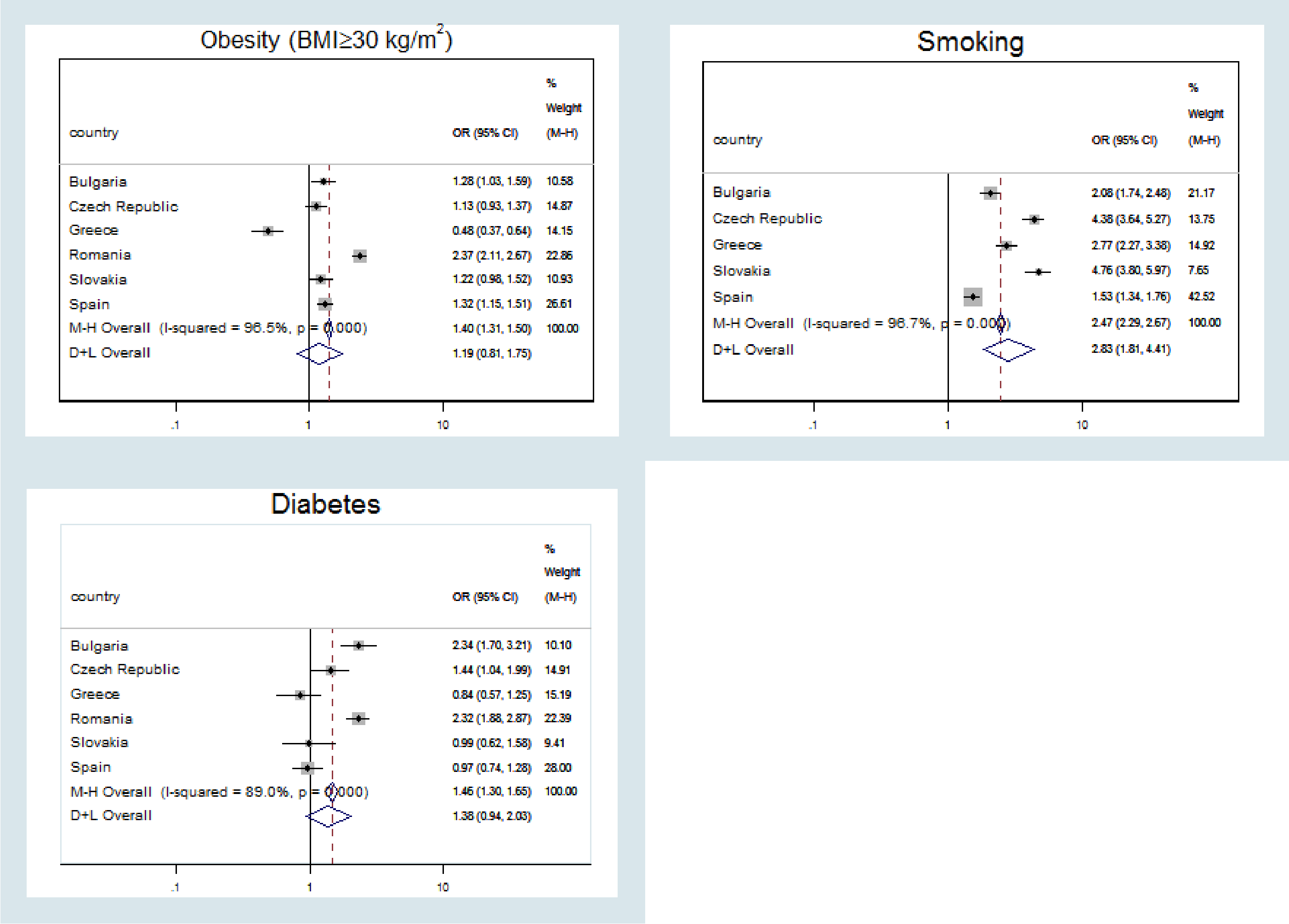

Figure S21. Forest plot of the association between a representative sample of ethnic Roma (participants in the European project "Health and the Roma Community, Analysis of the Situation in Europe" - UNDP/WB/EC) and higher odds ratio (OR) for the prevalence of three CVD risk factors (obesity, smoking and diabetes), compared to a representative sample of the majority population (the Eurostat data). Results are stratified by country.

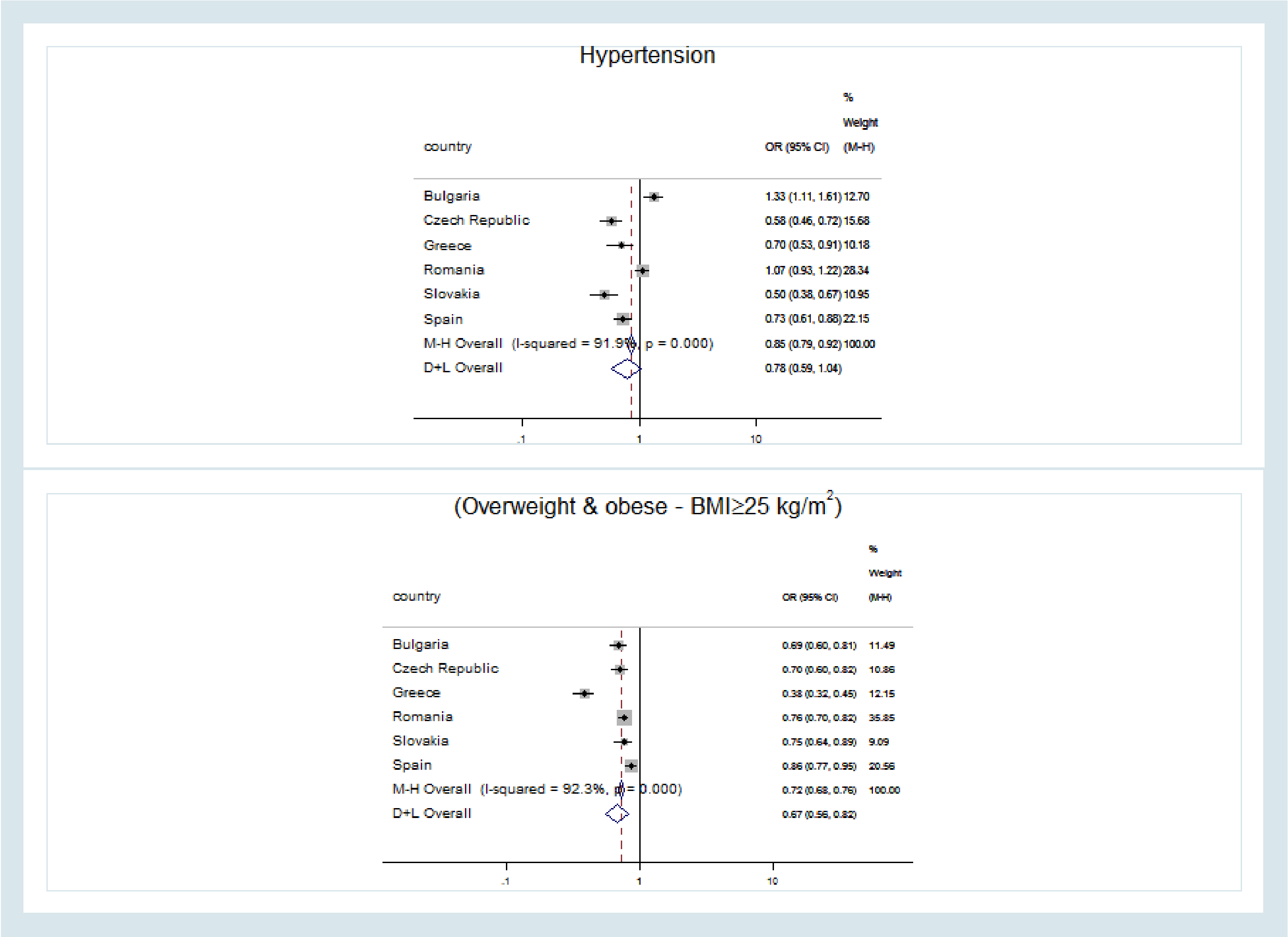

Figure S22. Forest plot showing an association between a representative sample of ethnic Roma (participants in the European project “Health and the Roma Community, Analysis of the Situation in Europe” - UNDP/WB/EC) and lower odds ratio (OR<1) for the prevalence of BMI≥25.00 kg/m^2 and hypertension, compared to a representative sample of the majority population (the Eurostat data). Results are stratified by country.
